# Adaptive and nonadaptive plasticity in changing environments: Implications for sexual species with different life history strategies

**DOI:** 10.1002/ece3.7485

**Published:** 2021-04-04

**Authors:** Daniel Romero‐Mujalli, Markus Rochow, Sandra Kahl, Sofia Paraskevopoulou, Remco Folkertsma, Florian Jeltsch, Ralph Tiedemann

**Affiliations:** ^1^ Evolutionary Biology/Systematic Zoology University of Potsdam Potsdam Germany; ^2^ Plant Ecology and Nature Conservation University of Potsdam Potsdam Germany; ^3^ Foundation, Zoology Institute University of Veterinary Medicine Hannover Hannover Germany; ^4^ Berlin‐Brandenburg Institute of Advanced Biodiversity Research (BBIB) Berlin Germany; ^5^ Biodiversity Research/Systematic Botany Institute of Biochemistry und Biology University of Potsdam Potsdam Germany; ^6^ Faculty of Life Sciences School of Zoology Tel Aviv University Tel Aviv Israel; ^7^ Evolutionary Adaptive Genomics University of Potsdam Potsdam Germany

**Keywords:** developmental canalization, environmental change, genetic accommodation, Individual‐based models, limits, many‐to‐one genotype–phenotype map, noise color, phenotypic plasticity, reaction norms, stochastic fluctuations

## Abstract

Populations adapt to novel environmental conditions by genetic changes or phenotypic plasticity. Plastic responses are generally faster and can buffer fitness losses under variable conditions. Plasticity is typically modeled as random noise and linear reaction norms that assume simple one‐to‐one genotype–phenotype maps and no limits to the phenotypic response. Most studies on plasticity have focused on its effect on population viability. However, it is not clear, whether the advantage of plasticity depends solely on environmental fluctuations or also on the genetic and demographic properties (life histories) of populations. Here we present an individual‐based model and study the relative importance of adaptive and nonadaptive plasticity for populations of sexual species with different life histories experiencing directional stochastic climate change. Environmental fluctuations were simulated using differentially autocorrelated climatic stochasticity or noise color, and scenarios of directional climate change. Nonadaptive plasticity was simulated as a random environmental effect on trait development, while adaptive plasticity as a linear, saturating, or sinusoidal reaction norm. The last two imposed limits to the plastic response and emphasized flexible interactions of the genotype with the environment. Interestingly, this assumption led to (a) smaller phenotypic than genotypic variance in the population (many‐to‐one genotype–phenotype map) and the coexistence of polymorphisms, and (b) the maintenance of higher genetic variation—compared to linear reaction norms and genetic determinism—even when the population was exposed to a constant environment for several generations. Limits to plasticity led to genetic accommodation, when costs were negligible, and to the appearance of cryptic variation when limits were exceeded. We found that adaptive plasticity promoted population persistence under red environmental noise and was particularly important for life histories with low fecundity. Populations producing more offspring could cope with environmental fluctuations solely by genetic changes or random plasticity, unless environmental change was too fast.

## Graphical abstract

This study shows that adaptive plasticity promoted population persistence under red noise stochasticity and was particularly important for life histories with low fecundity. Populations producing more offspring could cope with environmental fluctuations solely by genetic changes or random plasticity, unless environmental change was too fast. Adaptive plasticity through flexible interactions of the genotype with the environment led to (a) smaller phenotypic than genotypic variance in the population and the coexistence of polymorphisms, (b) many‐to‐one genotype–phenotype map, and (c) the maintenance of higher genetic variation—compared to linear reaction norms and genetic determinism—even when the population was exposed to a constant environment for several generations.

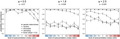

## 1 INTRODUCTION

A prevailing challenge in ecology and evolutionary biology is to understand and predict species’ responses to environmental change, such as climate change (Chevin et al., 2010; Gonzalez et al., 2013). Populations of different species are expected to respond to these changes by adaptation to novel local environmental conditions, shifts in their distributional range while tracking their preferred niche, or local extinction (Franks et al., 2014; Wiens, 2016). Particularly, when movement opportunities are constrained, populations are expected to either go extinct or to cope with novel conditions through adaptation by genetic changes, or phenotypic plasticity. Phenotypic plasticity is defined as the property of organisms sharing the same genotype to produce different phenotypes, often in response to the local environment (Pigliucci, 2005; Reusch, 2014; Sommer, 2020). It constitutes the nonheritable part of phenotypic variation and includes acclimation, developmental plasticity, behavioral flexibility, learning, maternal effects, epigenetics, and random noise (West‐Eberhard, 2003).

Among other factors, evolutionary rescue depends considerably on demographic properties and the generation time of a species (Bell, 2013; Chevin et al., 2010). Evolutionary rescue occurs when adaptive evolution allows a population to persist environmental conditions otherwise lethal for its ancestor (Bell, 2013; Klausmeier et al., 2020). Thus, organisms that do not reproduce often or produce relatively few progeny are expected to be vulnerable to extinction under environmental change due to their lower supply of beneficial mutations (Botero et al., 2015). Life history strategies where a genetic response is limited are expected to buffer fitness loss in a novel environment through plastic responses, which are generally faster (Botero et al., 2015; Lande, 2009). Using individual‐based models, different life history strategies have been compared for their ability to adapt to the local environment through genetic changes (e.g., Björklund et al., 2009). In contrast, the role of plasticity—particularly, the role of different types of phenotypic plasticity—for persistence under environmental change, has not yet been thoroughly investigated relative to different life history strategies. For example, in species with similar generation time, those with relatively high fecundity may rely less on plasticity as compared to species with clutch size limited to few offspring. Though it was not the focus of their work, Björklund et al. (2009) observed in their model that, all else equal, r‐like life history strategies persisted under environmental change the longest (as compared to other life history strategies) under scenarios of low heritability in which most variability of the phenotypic trait was developed randomly (random plasticity).

Phenotypic plasticity has long been considered important for organisms experiencing fluctuating environmental conditions (Scheiner, 1993; Via et al., 1995). Yet, the modeling of phenotypic plasticity has not been a straightforward task, since some features found in empirical research remain elusive to current approaches (e.g., limits to plasticity, Murren et al., 2015; many‐to‐one genotype–phenotype maps, Wagner, 2005). Furthermore, it remains elusive whether the process of genetic accommodation (genes as followers) is expected or not in nature, and whether some traits or taxa are particularly prone to it (Levis & Pfennig, 2020; Scheiner et al., 2017, 2020; Schlichting & Wund, 2014). The process of genetic accommodation occurs when phenotypic variants that are environmentally induced, become genetically determined by natural selection (Kulkarni et al., 2017; Schlichting & Wund, 2014; West‐Eberhard, 2003). On the other hand, changes in environmental conditions expose cryptic genetic variation that has been otherwise phenotypically silent. Cryptic variation refers to genetic variation that normally has little or no effect on phenotypic variation, but that under atypical conditions generates phenotypic variation (Paaby & Rockman, 2014). However, it is unclear whether cryptic genetic variation is the result of exposed hidden developmental programs that evolved from past selection or whether it is a manifestation of limitations of the developmental system of each organism (i.e., limits of plasticity) or a combination of both (Laland et al., 2015; Parsons et al., 2020).

This study presents an individual‐based eco‐evolutionary model of populations of sexual species with different life histories, experiencing scenarios of various rates of directional environmental change and different types of environmental stochasticity (noise color), and focuses on so far understudied aspects of plasticity. We specifically test for the effect of assuming (a) limits to plasticity and developmental flexibility of the plastic response on attributes of the population (e.g., genotypic, phenotypic variances) and ecological outcomes (probability of persistence), and (b) evaluating the performance of different types of plasticity and life histories under various environmental change scenarios (e.g., we ask what type of plasticity favors population persistence under a particular life history/change scenario?). This paper deals with organisms experiencing rapid environmental change and hence does not consider the evolution of plasticity itself. Assuming limits to the plastic response may add realism to the modeling of plasticity. For example, when costs of plasticity are negligible, as it seems to be the case for the majority of organisms (Murren et al., 2015), a model assuming perfect sensing and no limits may arrive at perfectly adapted phenotypes under all circumstances, while in nature, the plastic response is limited to a defined range of environmental conditions, beyond which perfect plasticity is physiologically impossible (Wiesenthal et al., 2018). Limits to plasticity occur when plasticity is unable to produce an “optimum” trait, even in the presence of enough resources or low cost, due to, for example, unreliable information, a time lag between the environmental change and the phenotypic response (DeWitt et al., 1998), or due to physiological limits or other constraints of the plasticity mechanism (e.g., of the underlying gene regulatory network). Furthermore, plasticity can result—at least for quantitative traits—from a complex relationship between genotype and phenotype, with the developmental system responding flexibly to internal (genotype) and external inputs (environment) (Laland et al., 2015). To this end, we compare linear reaction norms with alternative plasticity types, including a flexible developmental system. As a consequence, multiple genotypes can have the same phenotype and are mutationally interconnected (many‐to‐one genotype–phenotype map, Aguilar‐Rodríguez et al., 2018; Ahnert, 2017; Wagner, 2008). This assumption leads to new insights into the origin of cryptic genotypic variation, genetic accommodation, and the maintenance of genetic variation in natural populations.

From an ecological perspective, the evaluation of different forms of stochastic environmental conditions is important since the type (i.e., the color) of the environmental noise differently affects population extinction risk (Schwager et al., 2006; Mustin et al., 2013). Colored environmental noise arises when fluctuations of climatic variables such as temperature differ with regard to their serial autocorrelation between consecutive time units (typically years; Björklund et al., 2009; Laakso et al., 2001, 2004; Schwager et al., 2006). For instance, Mustin et al. (2013) found that extinction risk is expected to be high for populations experiencing directional climate change and inhabiting climates with reddish (i.e., positively autocorrelated) stochasticity. However, they did not consider scenarios of negatively autocorrelated stochasticity (blue noise), nor the effect of plasticity on population persistence. How such environmental stochasticity may promote the degree and mode of plastic responses has received less attention.

## 2 METHODS

To study the effect of adaptive and nonadaptive phenotypic plasticity on population persistence under scenarios of environmental change, we developed an eco‐evolutionary individual‐based model (IBM) of a geographically isolated panmictic population of a sexual species with nonoverlapping generations experiencing stochastic directional climate change. The focus was on studying the ability of a population to adapt to its local environment (no migration was possible). This modeling setup could resemble a fish population inhabiting a lake, or a plant or animal population inhabiting a highly fragmented environment where movement opportunities are constrained. Populations could differ in fecundity and intrinsic population dynamics (different life history strategies). The model also allows for different forms of environmental stochasticity or noise color: uncorrelated white noise typical for terrestrial locations; positively autocorrelated red noise, which had been found in coastal and marine habitats, Vasseur & Yodzis, 2004; and negatively autocorrelated blue noise. Blue noise is less common, but recent evaluations of climate spectral exponents suggest that temperature has turned bluer (i.e., tends toward more negatively autocorrelated stochasticity) in most continents in the last century (García‐Carreras & Reuman, 2011).

**FIGURE 1:**
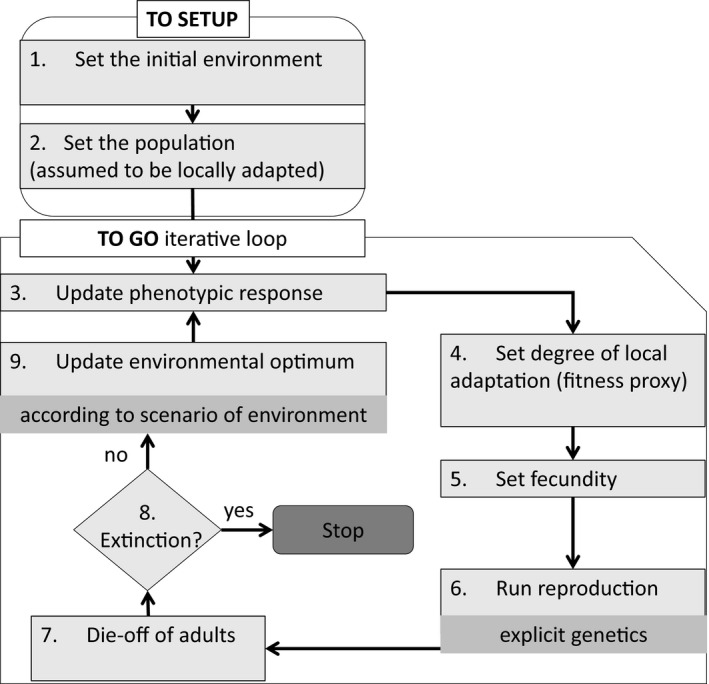
Flowchart diagram of the sequence of model operations

The model was created using the freely available software platform NetLogo 6.0.2 (Wilensky, 1999) and is available for download from https://github.com/danielrm84/PanModel33. A full description of the model that follows the ODD (Overview, Design, concepts, and Details) protocol (Grimm et al., 2006, 2010) can be found in Appendix A. Below, only model features that were used in this study are explained. The sequence of model operations was as follows: set initial environment and population (assumed to be locally adapted), update phenotypic response, check for degree of adaptation (as fitness proxy), computation of fecundity, reproduction of adults, inheritance, die‐off of adults, check for extinction, and update of the environmental state before repeating the loop (Figure 1).

### 2.1 Environment

**FIGURE 2:**
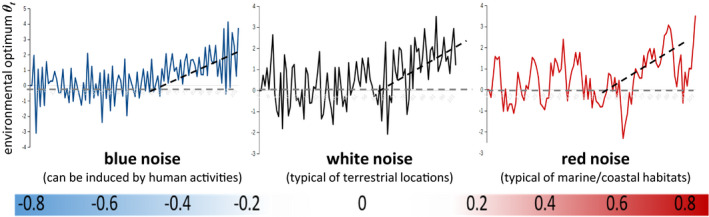
Example of scenarios of directional climate change and environmental stochasticity as simulated in the model. These scenarios resemble fluctuations of climatic variables, such as temperature, as measured for different kinds of habitats. Different forms of stochastic fluctuations (noise color) of the environmental optimum (*θ_t_*) were simulated. They differ in their temporal autocorrelation, that is, no autocorrelation (white), negative autocorrelation (blue), or positive autocorrelation (red). The dashed lines illustrate different rates of directional climate change (no change *η = 0*, slow to rapid change, scenarios of *η > 0*). Different scenarios of *η* were explored per scenario of colored noise (environmental autocorrelation). The color bar shows the range of values explored for the level of autocorrelation (*α*, see methods)

The environment imposed a phenotypic optimum *θ_t_* (hereafter, environmental optimum) which could change at constant speed every generation depending on the simulated scenario of environmental change. Thus, *θ_t_ = θ_0_ + η t* determined the directional trend of the optimum *θ_t_* in a deterministic environment (no stochasticity). The parameter *θ_0_* was the initial environmental optimum (when *t = 0*), and *η* was the rate of environmental change. By varying the parameter *η,* we simulated different scenarios of directional climate change (e.g., no change, slow, medium, rapid climate change). Stochastic colored noise around *θ_t_* was implemented to simulate different scenarios of environmental stochasticity (Figure 2). This method has been recommended for the simulation of directional climate change scenarios (Kopp & Matuszewski, 2014; Vincenzi, 2014).

Stochasticity according to colored noise was implemented such that the environmental optimum was determined by θ_t_ = θ*_t_ + ϕ_t,_ where θ*_t_ gave the directional trend of the mean environmental optimum as specified above and *ϕ_t_ = αϕ_t−1_ + βξ_t_* the environmental stochasticity. The parameter *α* governed the level of environmental autocorrelation and therefore allowed for different forms of stochasticity or noise color as in Björklund et al. (2009): *−1 < α < 0*, blue noise; *α = 0*, white noise, and *0 < α < 1*, red noise (Figure 2). Several scenarios of noise color (values of *α*) were explored, ranging from negatively autocorrelated environmental conditions or blue noise over uncorrelated (white noise) to positively autocorrelated environmental conditions or red noise (see Table 1). The parameter *β = σ* $\sqrt{1‐\alpha^{2}}$ was the adjusted environmental variance for all degrees of autocorrelation, as in (Schwager et al., 2006), and *σ^2^ = 1* was the environmental variance. The parameter *ξ_t_* was a random value, normally distributed with zero mean and unity as variance.

**TABLE 1:** Parameter values and description

Parameter	Value	Description
*K*	1,000	Carrying capacity
*γ*	2.2	Strength of selection. Moderate selection (Björklund et al., 2009)
*σ^2^*	1	Variance of the stochastic environment
*θ_0_*	0	Initial environmental optimum
*η*	0, 0.01, 0.02, 0.025, 0.03, 0.04	Rate of environmental change
*α*	−0.8, −0.6, −0.4, −0.2, 0, 0.2, 0.4, 0.6, 0.8	Level of environmental autocorrelation (scenarios of stochasticity)
*ψ*	0.5, 1.0, 1.8, 2.5	Density dependence effect (life history strategy) (as in Björklund et al., 2009)
*z*	evolving trait	Ecological phenotype
*b*	0.5	Slope of the linear reaction norm
*VG*	0.2	Initial genetic variance present in the population (Vincenzi, 2014)
*L*	1	Number of loci per evolving trait
*μ*	10^–3^, 10^–4^	Mutation rate^a^
*MV*	0.2	Variance of the distribution of mutations fitness effect size (Vincenzi, 2014)
*t*	100, 250	Time limit per simulation, in generations
*sd*	10 and 20% the value of *K*	Standard deviation of the stochastic carrying capacity

^a^ The value of the mutation rate was picked according to results of a simulation model on the evolution of the mutation rate after 300 generations of a population experiencing stochastic environmental conditions (no climate change, and no plasticity, Romero‐Mujalli et al., 2019a). In addition, this value is within the range of mutation rates used in other simulation models (reviewed in Romero‐Mujalli et al., 2019b).

### 2.2 The population

Individuals in the population were characterized by sex, stage (whether adult or juvenile), degree of adaptation (fitness proxy, depends on the ecological phenotype and the environment), fecundity (influenced by the degree of adaptation and the population density), and an ecological phenotype (evolving trait). The ecological phenotype *z* had its genetic component *a* defined by *L* diploid loci with additive effects and its environmental component *e* determining the contribution of phenotypic plasticity in the expression of the genotype *a*. Thus, *z_i_ = a_i_ + e*, was the ecological phenotype of individual *i*, where *a_i_* was the sum of allelic values at the adaptation locus. Thereby, the variance of *z* determined the phenotypic variance in the population, and the variance of *a* represented the genetic variance. We assumed one diploid locus affecting the phenotypic trait. At the beginning of the simulation, the population was composed of *N* individuals at carrying capacity (*N = K = 1,000*) and locally adapted. This means that for each individual organism, alleles coding for its ecological phenotype were drawn from a normal distribution with mean equal to the environmental optimum *θ_0_* and variance *V = VG/2*, where *VG = 0.2* was the initial genetic variance present in the population.

### 2.3 Phenotypic plasticity

**FIGURE 3:**
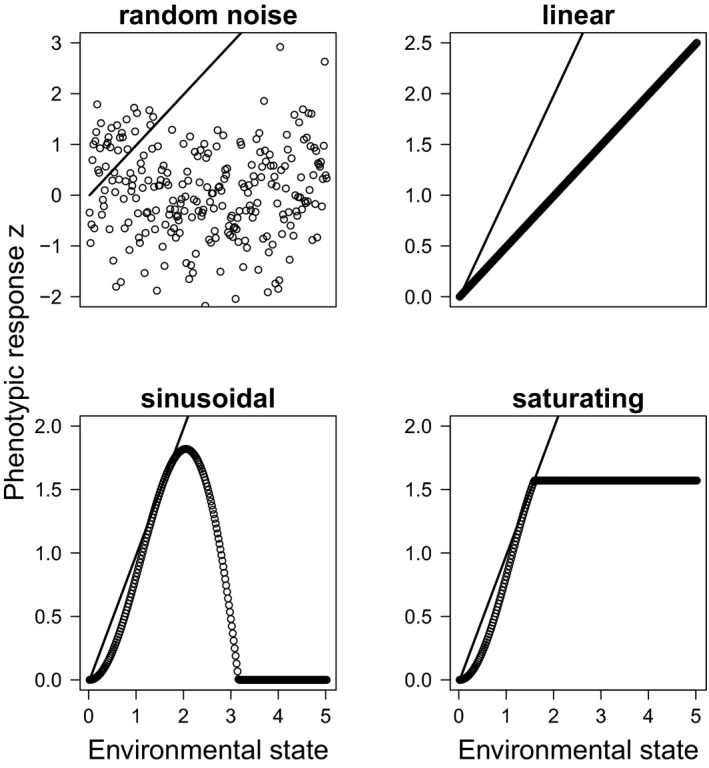
Forms of nonadaptive (random) and adaptive (linear, sinusoidal, saturating) phenotypic plasticity implemented in the model. The black line indicates the moving phenotypic optimum *θ_t_* as given by the environment. Empty circles show the phenotypic response of the organism

Because we wanted to compare traditional approaches with alternative ones imposing limits to plasticity (objective 1, see Introduction), four different types or scenarios of phenotypic plasticity were implemented (Figure 3): random noise, linear reaction norm, sinusoidal reaction norm, and saturating reaction norm. In the model, phenotypic plasticity affected the environmental component *e* of the ecological phenotype. Random noise has been the most common method in eco‐evolutionary IBMs for the representation of an environmental effect on the development of the phenotypic trait (Romero‐Mujalli et al., 2019b). In this model, we consider random noise as part of nonadaptive phenotypic plasticity. In contrast, the other three methods were considered adaptive since they enabled phenotypic adjustments in the direction of the phenotypic optimum *θ_t_*. Thus, we could compare the effect of nonadaptive and adaptive phenotypic plasticity on population persistence. In the model, random plasticity assumed *e* to be random and normally distributed with zero mean and variance *VE = σ^2^*, the environmental variance.

For the modeling of adaptive phenotypic plasticity, we considered three approaches: the linear reaction norm without a limit, which is the most common approach in the literature (Chevin et al., 2010; Lande, 2014; Romero‐Mujalli et al., 2019b), and two other approaches that account for limits to phenotypic plasticity (Figure 3). In the literature, most research has focused on costs of plasticity, and very little on its limits (Murren et al., 2015).

In our model, the linear reaction norm defined the environmental component as *e = bθ_t_*, where *b* is the slope or degree of plasticity and *θ_t_* is the environmental optimum at time *t* (in generations). Furthermore, the linear reaction norm was assumed to be shallower (*b = 0.5*) and in the direction of the phenotypic optimum *θ_t_*. Hence, the linear plastic response was adaptive, but that there was a misfit arising from an imperfect development of the trait (Ashander et al., 2016; Chevin et al., 2010; Lande, 2009). Note that assuming *b = 1* will lead to “perfect plasticity.” It is important to mention that perfect sensing of the environment was assumed for all scenarios of adaptive phenotypic plasticity. Reliability of sensing, that is, the correlation between sensed environmental cues and environmental variables affecting fitness, has been investigated by Reed et al. (2010), Ashander et al. (2016), and Ergon and Ergon (2016), but was beyond the scope of our work.

The two other methods, saturating and sinusoidal, were designed based on observations from stress tolerance responses for some physiological traits (Araújo et al., 2014, 2016; Jordan & Deaton, 1999; Solan & Whiteley, 2016; Wiesenthal et al., 2018) and on behavioral traits. Their plastic response was assumed to be developmentally flexible (Laland et al., 2015), relying on feedback with the environment, and in the direction of the environmental optimum (adaptive). This means that they allowed for stable functioning of the phenotype (close to the optimum) despite the variation at the genetic level. Furthermore, in contrast to linear reaction norms, they enable multiple genotypes to have the same phenotype, which is a widespread feature of genotype–phenotype maps (Ahnert, 2017; Aguilar‐Rodríguez et al., 2018; Payne & Wagner, 2019). These methods differed from each other only in the condition that determined the phenotype produced when the limit is exceeded and were implemented as follows:

*e = ΩΔE*, where *Ω* was always positive and shaped the plastic response. It was given by *Ω = sin(*│*ΔE*│*)*. The term *ΔE* indicated the amount of change with respect to the reference environment in which plasticity is not needed and was defined as *ΔE = θ_t_ – a*. The parameter *a* was the genetic component of the phenotype.

For sinusoidal phenotypic plasticity, if the argument of the sine function was greater than *π*, the environmental component *e* was set to *0* (the organism fails to develop a plastic response). An example could be snails subject to salinity stress. If the change is too large (compared to the reference environment where plasticity is not needed), snails fail to develop enough physiological response to counter and balance osmotic pressure (e.g., Jordan & Deaton, 1999; Wiesenthal et al., 2018).

For saturating phenotypic plasticity, if the argument of the sine function was greater than *π/2*; the term *ΔE* was set to *ΔE = π/2* such that a maximum response was reached (saturation). This could resemble plant species exposed to different light conditions. After some point of increasing light intensity, a maximum thickness will be reached, and the plant's leaves would not grow any thicker (Wilson & Cooper, 1969).

According to the sequence of model operations (Figure 1), the plastic response always followed the environmental change. This implementation implies phenotypic traits being flexible enough such that adult individuals could still respond to environmental change. However, some characters cannot be further modified after a sensitive period early in life (constant characters, Lande, 2014; Romero‐Mujalli et al., 2019b), such that adults experiencing a new environment will no longer be able to adjust their matured phenotype. In order to evaluate the impact of the ontogenetic phase of plasticity (flexible or labile plasticity versus. fixed during ontogeny or constant characters after an early sensitive period) on population persistence, we also study the effect of phenotypic plasticity when the plastic response precedes the environmental change.

### 2.4 Degree of adaptation

After developing the phenotype, adult individuals in the population were subject to selection according to the following Gaussian function (Burger & Lynch, 1995):

$$w_{i}=e^{\frac{{‐(z_{i}‐\;\theta_{t})}^{2}}{2\gamma^{2}}}$$

where *w_i_* was the degree of adaptation (fitness proxy) of individual *i*, and *γ* was the width of the function (strength of selection). The closer the ecological phenotype *z_i_* was to the optimum *θ_t_*, the better the individual coped with the environmental conditions.

### 2.5 Fecundity and life history strategies

**FIGURE 4:**
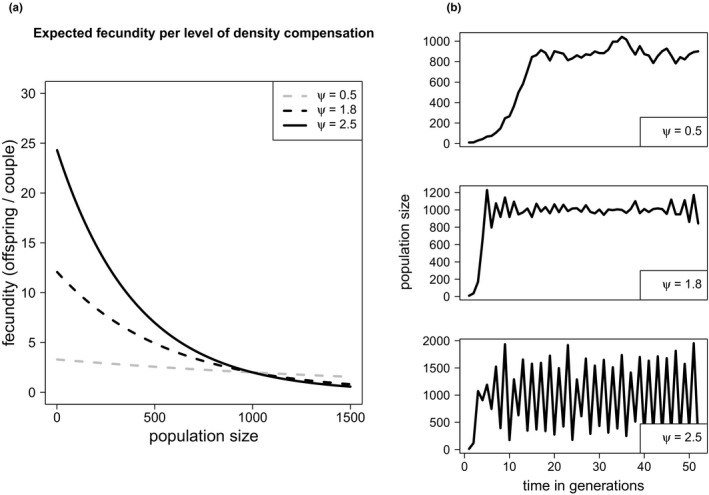
Life history strategies as implemented in the model. (a) Expected fecundity (*λ*) per reproductive couple for different values of population size (carrying capacity *K = 1,000*). When the population size is low, resources are plenty, and well‐adapted couples can contribute their best in number of offspring (fecundity) for the next generation. (b) Population dynamics (carrying capacity *K = 1,000*) per life history strategy (values of *ψ*) in a static environment (no climate change, no stochasticity)

The fecundity of individuals in the population was scaled according to their degree of adaptation after considering density dependence effects and follows a Ricker‐type model as in Björklund et al. (2009):

$$w_{i}^{\prime }=w_{i}e^{\psi \left(1‐\frac{N}{K}\right)}$$

where *w'i* was the fecundity of individual *i*, *N* was the population size, and *K* was the carrying capacity. The parameter *ψ* described the strength of the density dependence effect (also referred here as density compensation). The higher *ψ*, the stronger was the effect of increased population density *N*. Here, we implemented three levels of *ψ* as in Björklund et al. (2009): *0.5*, *1.8,* and *2.5*. These three values produced fundamentally different population dynamics and therefore cover a wide range of different outcomes due to *ψ* (Figure 4a and b).

### 2.6 Reproduction

Adult individuals mated randomly with others of opposite sex, with replacement for males only (i.e., lottery polygyny, males could participate in more than one reproductive event). The fecundity of the reproductive couple *λ* was equal to the sum of the scaled fitness of the partners *i*,*j*:

$$\lambda =w_{i}^{\prime }+w_{j}^{\prime }$$

Each couple produced a number of offspring randomly drawn from a Poisson distribution with expectancy *λ*.

### 2.7 Inheritance

After reproduction, each offspring inherited one allele from each parent. Only the genetic component *a* was inherited. Allele values of the inherited haplotype were picked randomly from the corresponding parental locus. Each haplotype mutated with probability *μ* of mutation per locus. In case that a mutation occurred, its effect was randomly drawn from a normal distribution with zero mean and variance equal to the effect size of mutations *MV*, which was an input parameter (Vincenzi, 2014). The assumption of a Gaussian distribution is consistent with analysis of mutation effects (Lynch & Walsh, 1998; Martin et al., 2006).

All adults died after reproduction (nonoverlapping generations), and the environmental state was updated before repeating the loop.

### 2.8 Simulation experiments

With the model, we studied the effect of assuming limits to plasticity and developmental flexibility on attributes of the population (objective 1) and studied the effect of these types of plasticity (nonadaptive and adaptive phenotypic plasticity) on the persistence of populations with different life histories under scenarios of directional stochastic environmental change (objective 2). Probability of persistence was computed as the proportion of times a persistence event (or success) occurs from the total number of observations (the 100 replicates). Error bars were estimated from the binomial distribution. A total of 100 replicates lasting 250 generations each were performed for different combinations of rate of directional environmental change, degree of temporal autocorrelation (noise color), type of phenotypic plasticity (including genetic determinism), and density dependence effect (life history strategy) (see Table 1). To illustrate selected parameter values: Under no stochasticity, *η = 0.02* imposed a directional trend on the optimum phenotype of 0.02 phenotypic units per generation (or per year, assuming one generation a year). This means that after 250 generations of simulation time, the optimum *θ_t_* would have changed in five phenotypic units, and a population with no density compensation (i.e., *ψ = 1*) could avoid extinction via evolutionary rescue or some sort of phenotypic plasticity. To our experimental setup, the contribution of mutations was important. Otherwise, populations failed to cope with the changing environmental conditions (Figure S1, Appendix B: Supplementary material). It is important to highlight that, in our model, limits of plasticity were reached well before the simulation ended. Following the same example, an initially well‐adapted individual (*z_i_ = θ_0_*) reached its limits of plasticity (i.e., saturation, by saturating plasticity; decay in ability of plastic response, by sinusoidal plasticity) at *θ_t_ = 1.58*. This corresponds to generation *t = 80*.

To complement the findings on life history strategies of intermediate and strong density dependence effects, additional scenarios of rate of environmental change and of stochastic fluctuations in carrying capacity were implemented to unravel whether, in the model, life history strategies differently tolerate fluctuations in K. Specifically, for each time unit or generation, the value of carrying capacity was drawn from a normal distribution centered in *K* and variance *sd* ^2^, where *sd* was a percentage of *K*. For instance, if *K = 1,000*, the scenario of 10% of *K* means *sd = 100*. This is, around 70% of the cases, the value of carrying capacity that is expected to occur within the interval (*K – sd*, *K + sd*).

Simulation experiments were also evaluated under scenarios of lower mutation rate, and mutational effects according to the model of slightly deleterious mutations (Eyre‐Walker et al., 2002; Ohta, 1973; Romero‐Mujalli et al., 2019a), which imposed higher genetic constraints. The analysis of data and plotting was performed in r v3.5.2.

## 3 RESULTS

### 3.1 Implications of assuming limits and flexible development for the modeling of phenotypic plasticity

**FIGURE 5:**
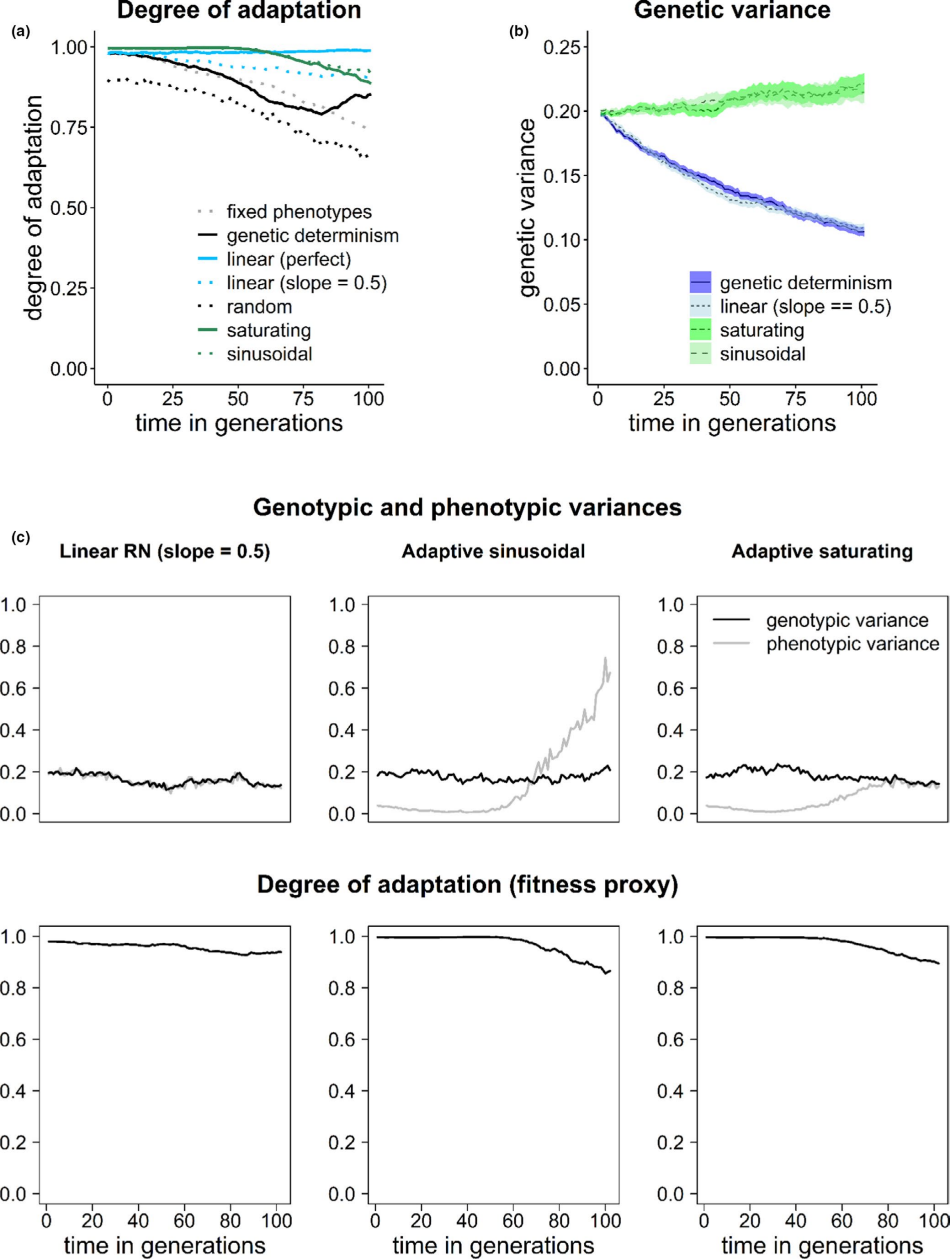
(a) Mean degree of adaptation (fitness proxy) in populations with and without adaptive phenotypic plasticity experiencing directional deterministic environmental change (rate of change *η = 0.03*, density dependence effect *ψ = 1.0*, mutation rate *µ = 0.001*). A scenario of no adaptation (*µ = 0*) was also simulated, for comparison. The simulation was run for 100 generations. (b) Genetic variance (average ± standard error from 100 replicates) present in a population experiencing constant environmental conditions (no climate change, no stochasticity; e.g., laboratory conditions) per scenario of phenotypic plasticity. (c) Time series of the genotypic and phenotypic variances present in a population experiencing deterministic directional environmental change (no stochasticity). Under the scenarios of adaptive sinusoidal and saturating phenotypic plasticity (upper panel), when the population is locally adapted (initial part), there is less phenotypic than genetic variation (*developmental canalization*, at the population level, Posadas & Carthew, 2014). Under the assumption of adaptive sinusoidal plasticity, as the environment changes and the population are pushed toward its limits of plasticity, cryptic genotypic variation arises, and the mean fitness (here, degree of adaptation) of the population reduces (lower panel). In linear reaction norms, the genotypic and phenotypic variances are directly proportional

Considering the degree of adaptation under directional deterministic environmental change (no stochasticity, typical of laboratory experiments), any form of adaptive phenotypic plasticity was of advantage over nonadaptive plasticity and absence of plasticity (i.e., genetic determinism) (Figure 5a). When the slope equals one, linear reaction norms yield perfect plasticity, while the “perfect match” of the phenotype with the environmental optimum depends on whether the limits are exceeded or not for those forms of plasticity that account for limits (Figure 5a).

Interestingly, adaptive sinusoidal and saturating plasticity led to stable functioning phenotypes (close to the optimum) despite the variation at the genetic level (*developmental canalization* at the population level, Posadas & Carthew, 2014) (Figure 5b,c). A consequence of this property is that they maintained higher genetic variance than the alternative methods even when the population was exposed to a constant environment (e.g., laboratory conditions) for several generations (Figure 5b). Additionally, in the model assuming a sinusoidal reaction norm, cryptic variation appeared in the population when it was pushed toward the limits (Figure 5c).

**FIGURE 6:**
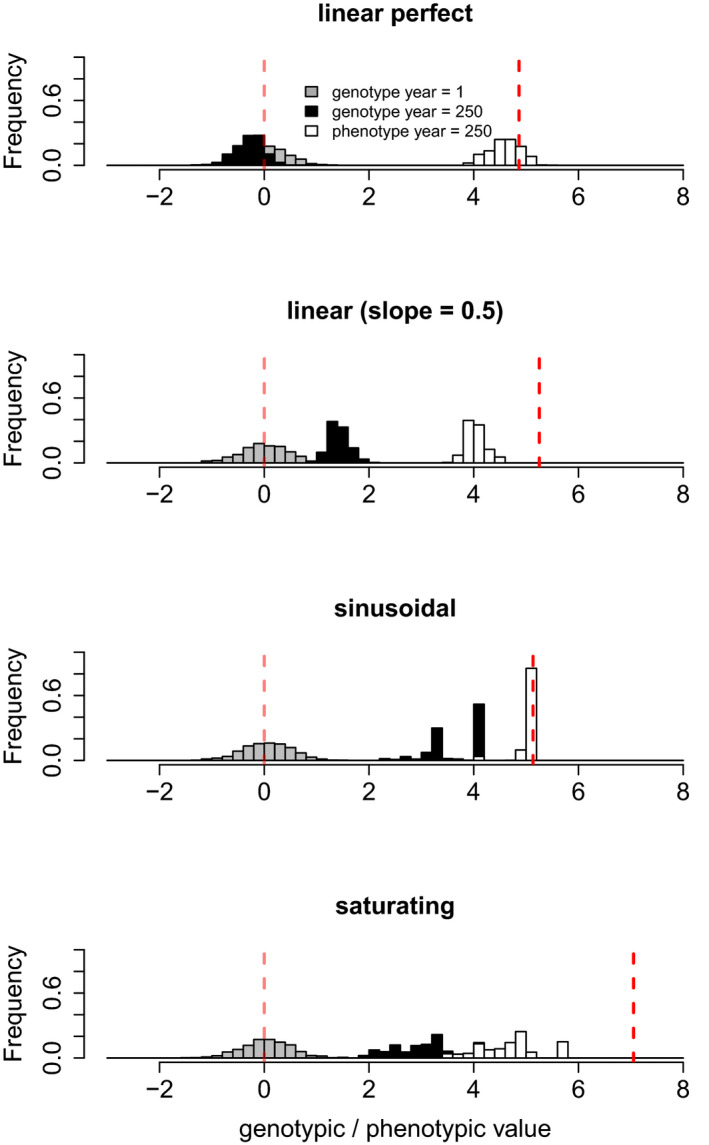
Genotypic and phenotypic distributions of a population experiencing directional stochastic environmental change (rate of change *η = 0.02*, level of environmental autocorrelation *α = 0*, white noise, density dependence effect *ψ = 1.0*, mutation rate *µ = 0.001*) under scenarios of adaptive phenotypic plasticity (linear perfect, linear imperfect, *slope = 0.5*, saturating, and sinusoidal plasticity). Gray, genotypic distribution at year 1, black, genotypic distribution at year 250; white, phenotypic distribution at year 250. The red vertical lines show the phenotypic optimum as given by the environment at the start (year 1) and end (year 250) of each simulation run. Note that each plot corresponds to an independent simulation run

Assuming limits to phenotypic plasticity led to genetic accommodation (genes as followers) in the absence of costs of plasticity (Figure 6). When plasticity is perfect or nearly perfect, however, resulting phenotypes closely followed the optimum with minimal to no genetic changes occurring during the simulated environmental change (Figure 6, linear reaction norms). Sinusoidal adaptive plasticity showed lower phenotypic than genotypic variation and corresponds to an example of developmental canalization at the population level (Figures 6 and 5c). Saturating phenotypic plasticity showed the manifestation of otherwise cryptic genotypic variation when the population was pushed toward its limits of plasticity (Figure 6).

### 3.2 Phenotypic plasticity: life histories under environmental change

**FIGURE 7:**
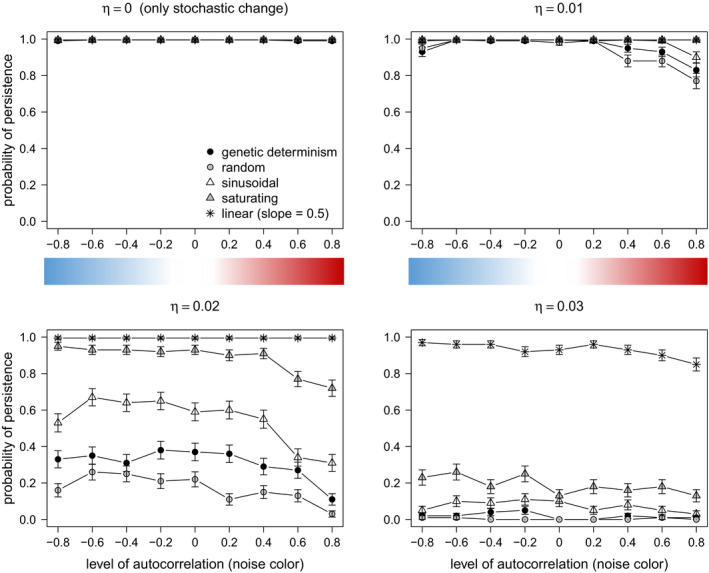
The effect of nonadaptive (random) and adaptive (linear, sinusoidal, and saturating) phenotypic plasticity on probability of persistence (100 replicates, 250 generations each) of a population in which breeding females are limited to produce relatively few offspring (*ψ = 0.5*, weak density dependence effect). The linear reaction norm had a slope *b = 0.5*. A scenario of genetic determinism (narrow sense heritability *h^2^ = 1*) was also simulated, for comparison. The color bar illustrates the color of the environmental noise

**FIGURE 8:**
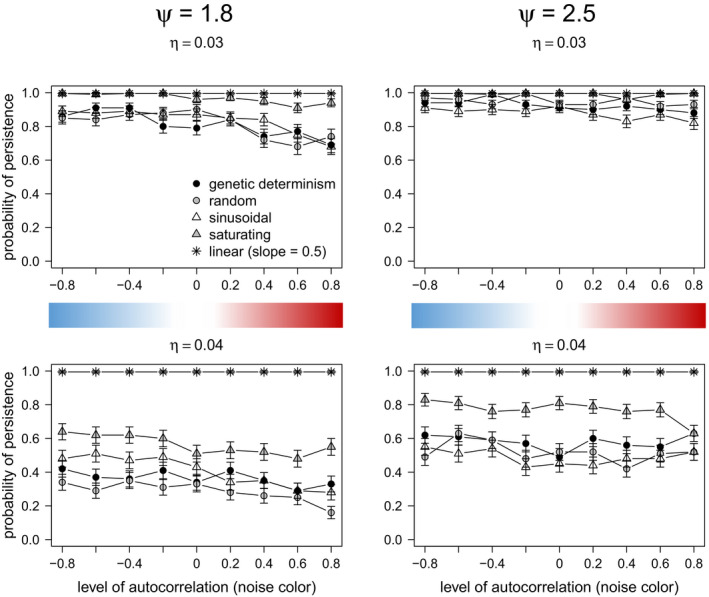
Effect of nonadaptive (random) and adaptive (sinusoidal, saturating, and linear) phenotypic plasticity on the probability of persistence (100 replicates, 250 generations each) of a population of intermediate and strong density dependence effect (*ψ = 1.8 and 2.5*, respectively). The linear reaction norm had a slope *b = 0.5*. A scenario of genetic determinism (narrow sense heritability *h^2^ = 1*) was also simulated. The color bar illustrates the color of the environmental noise. Scenarios of *η < 0.03* were not shown, because all treatments led to maximum probability of persistence

Under scenarios of weak density dependence effect (*ψ = 0.5*), in which breeding females in the population could produce relatively few offspring, adaptive phenotypic plasticity played a major role promoting persistence as compared to organisms with higher *ψ* (Figures 7, 8). Particularly for this life history strategy under slow climate change, adaptive phenotypic plasticity became of high importance promoting adaptation under positively autocorrelated environmental stochasticity (red noise). When the rate of change *η* was too rapid, adaptive phenotypic plasticity (particularly, linear reaction norm, and saturating plasticity) became advantageous for all simulated environmental conditions of noise color (Figure 7).

In contrast, results of strong and very strong density compensation (*ψ = 1.8 and 2.5*, respectively) always persisted under conditions of only environmental stochasticity (no directional climate change) and relatively slow to medium rate of environmental change (*η = 0, 0.01, 0.02*), regardless of the type of environmental stochasticity (noise color). For these life history strategies, genetic determinism and all forms of plasticity performed equally well (Figure 8).

Under scenarios of relatively rapid directional climate change, adaptive linear and saturating phenotypic plasticity performed the best for organisms of intermediate and high‐density dependence effects (*ψ = 1.8* and *ψ = 2.5*) (Figure 8, *η = 0.04*). The life history strategy with weak density compensation was not included in this analysis, since its populations always went extinct, except for the scenarios of linear and saturating plasticity. On the other hand, adaptive sinusoidal, nonadaptive random phenotypic plasticity, and genetic determinism showed similar performance across all scenarios of rapid rate of directional climate change (Figure 8).

**FIGURE 9:**
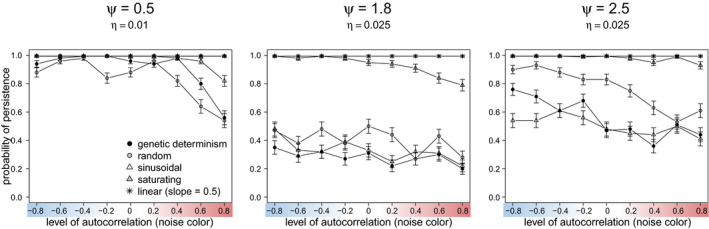
Relative importance of forms of nonadaptive (random) and adaptive (sinusoidal, saturating and linear) phenotypic plasticity affecting persistence of populations differing in levels of density compensation (*ψ*) and experiencing moderate rate of directional stochastic environmental change (*η = 0.01*, *ψ = 0.5*; *η = 0.025*, *ψ = 1.8* and *ψ = 2.5;* 100 replicates, 250 generations each) under scenarios of low mutation rate (*μ* = 10^–4^). A higher *η*, results for *ψ = 0.5* were not shown, because scenarios of random and sinusoidal plasticity, and genetic determinism (narrow sense heritability *h^2^ = 1*) went usually extinct. The color bar illustrates the color of the environmental noise

**FIGURE 10:**
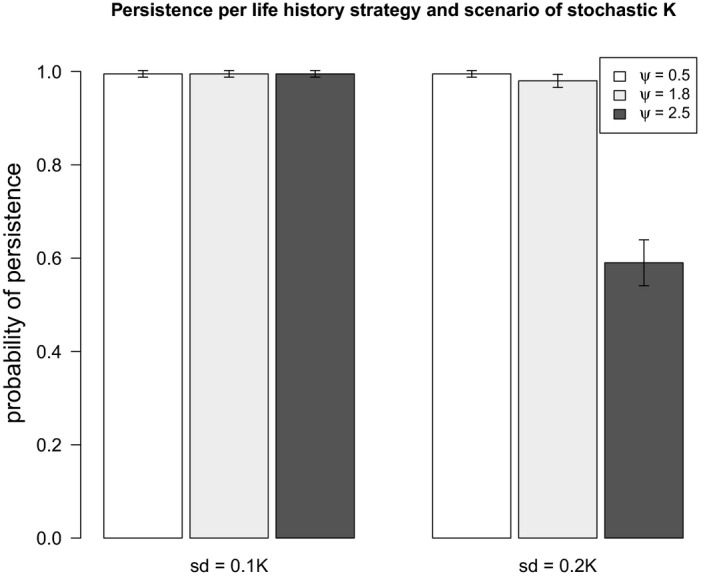
Probability of persistence (100 replicates, 250 generations each) per life history strategy (levels of *ψ*) under stochastic fluctuations in both, the carrying capacity (*sd = 10* and *20%* of *K*) and the phenotypic optimum (white noise, no directional climate change)

The performance of random plasticity was notably prominent for organisms of intermediate and high‐density dependence effects under scenarios of lower mutation rates (*μ* = 10^–4^, Figure 9). Similar results were observed when considering *μ* = 10^–3^ and mutational effects according to the model of slightly deleterious mutations (Eyre‐Walker et al., 2002; Ohta, 1973), as in Romero‐Mujalli et al. (2019b) (Figure S2, Appendix B: Supplementary material). When genetic mechanisms producing novel variation are somehow constrained, these organisms can cope with changing environmental conditions relaying on nonadaptive phenotypic plasticity only (Figure 9). This ability was not observed for organisms of weak density compensation, typical of large mammals and some bird species. However, populations of organisms with strong density compensation (*ψ = 2.5*) were considerably more vulnerable to extinction due to stochastic fluctuations in the carrying capacity (Figure 10).

When the plastic response precedes the environmental change (i.e., change after a sensitive period of plastic trait development), the dynamic can considerably change. For example, for those life history strategies of weak density compensation (*ψ = 0.5*) under blue noise, adaptive forms of phenotypic plasticity showed the lowest persistence (Figure S3, Appendix B: Supplementary material). Under this scenario, genetic determinism and random plasticity performed the best in terms of probability of persistence (Figure S3, Appendix B: Supplementary material). Whether plastic responses precede or follow the environmental change seems to have little impact under life history strategies with stronger density compensation (Figure S3, Appendix B: Supplementary material).

## 4 DISCUSSION

The objectives of this study were as follows: (a) to evaluate the effect of assuming plasticity as a developmentally flexible phenotypic response with limits and (b) to assess the relative importance of adaptive and nonadaptive plasticity for populations of sexual species with different life history strategy experiencing scenarios of directional climate change and environmental stochasticity (noise color). The simulated environmental conditions, though simplified as in every model, mimic realistic expected scenarios of environmental climate change (Björklund et al., 2009; Kopp & Matuszewski, 2014; Vincenzi, 2014). Our results show that the relative importance of phenotypic plasticity varies among life history strategies. Furthermore, they show that the advantage of nonadaptive and adaptive forms of phenotypic plasticity on population persistence depends on the type of environmental stochasticity (noise color) and the rate of directional climate change. In addition, the advantage of adaptive and nonadaptive forms of plasticity depends on whether plasticity precedes or follows the environmental change. Finally, assuming limits to plasticity leads to genetic accommodation (in the absence of costs to plasticity) and to the appearance of cryptic genetic variation when limits are exceeded. Moreover, assuming plasticity as the result of genotypes responding flexibly to feedback from their environment leads to developmental canalization at the population level (Posadas & Carthew, 2014), many‐to‐one genotype–phenotype map (Aguilar‐Rodríguez et al., 2018; Wagner, 2008) promoting the coexistence of polymorphism, and higher maintained genetic variation, potentially increasing evolvability, as compared to traditional approaches (e.g., linear reaction norms).

### 4.1 On the limits and the model implementations of adaptive phenotypic plasticity

Our work explicitly accounts for limits to plasticity. Though a thorough analysis of limits to plasticity was beyond the scope of this study, there are some aspects worth consideration. Linear reaction norms have no limits, and their evolution can theoretically result in organisms displaying perfectly adapted phenotypes under all environmental contexts, if no cost is imposed, which is unrealistic. Plastic responses certainly have energetic demands (i.e., costs), but also limits to plasticity—in the absence of costs—can result from the properties and characteristics of the underlying machinery producing the phenotypic response. Therefore, linear adaptive phenotypic plasticity can overestimate the probability of persistence. In our simulations, this was prevented by having a slope of 0.5 instead of 1. Even so, linear adaptive phenotypic plasticity had the best performance (in terms of increasing population persistence) in comparison to the other methods of adaptive plasticity. In the model, it took approximately 80, 60, and 40 generations or years to cross the limits of plasticity for a rate of directional change of 0.02, 0.03, and 0.04, respectively. Thus, for the population to persist the 250 generations under such scenarios of environmental change, genetic changes need to necessarily follow to enable survival beyond plasticity limits (Figure 6). Only linear adaptive phenotypic plasticity, as simulated in the model, could—for some life history strategies, *ψ = 1.8* and *2.5*—lead to persistence of the population with minimal, if any, genetic changes. Thus, the existence of limits to plasticity (without the consideration of costs; that is, when costs are negligible, see Murren et al., 2015) can lead to a transition from an “environmentally induced” to a “genetically encoded” state of a trait (genetic accommodation, Sommer, 2020).

Previous theoretical studies show that plasticity is of advantage when costs are low (Chevin et al., 2010; Lande, 2014; Scheiner et al., 2020). Furthermore, Scheiner et al. (2017) found that assuming phenotypic plasticity with no limitations and minimum costs, typical of linear reaction norms, can prevent genetic accommodation. We add that, given that limits to plasticity can differ among traits and taxa, the prevalence of genetic accommodation will also differ between traits and species. Specifically, genetic accommodation will more likely take place in those traits/species with narrower limits of plasticity. When plasticity is limited, our model suggests that populations go extinct, unless genes follow the plastic response in order for the population to sustain the environmental change. Demographic and genetic properties, the type of environmental fluctuations, and whether the trait is labile or constant play an important role in enabling this genetic response (see below, discussion 4.2). In contrast, species with “no apparent limit” to plasticity can adjust their phenotype through phenotypic plasticity with minimal genetic changes.

Our model also shows that when the environmental change pushes toward the limits of plasticity, cryptic genotypic variation may arise (Figure 5c). This means higher phenotypic than genotypic variance, a ratio that could be measured in nature. This feature comes along with a reduction in mean fitness of the population (in the model, degree of adaptation) and could potentially be used as an early warning signal for the inability of a population to sustain environmental change (Boettiger et al., 2013).

In our model, forms of phenotypic plasticity with limits are assumed to enable diverse functional responses through flexible interactions of the genotype with the environment (Laland et al., 2015; van Gestel & Weissing, 2016). This assumption leads to, first, stable functioning of the phenotype (close to the optimum) despite the variation at the genetic level (*developmental canalization* at the population level, Posadas & Carthew, 2014) (Figure 5). Second, multiple genotypes can have the same phenotype (many‐to‐one genotype–phenotype map, Aguilar‐Rodríguez et al., 2018; Ahnert, 2017) (Figure 5c), thus enabling coexistence of polymorphism when different genotypes have the same fitness. Third, genotypes with the same phenotype are mutationally interconnected, such that small mutations can transform these genotypes into one another without altering the phenotype (robustness) or lead to new phenotypes (evolvability), at the same time (Aguilar‐Rodríguez et al., 2018; Ahnert, 2017; Payne & Wagner, 2019; Wagner, 2008). Moreover, it can act as a mechanism that maintains genetic variation, potentially increasing evolvability (van Gestel & Weissing, 2016). In our implementation of adaptive plasticity, this is evidenced by the sinusoidal and saturating plasticity scenarios resulting in the highest maintained genetic variation—higher than linear reaction norms and genetic determinism—even when the population is exposed to a constant environment (e.g., laboratory conditions) for several generations (Figure 5b). This agrees with observations on the maintenance of adaptive capacity in populations (often clonal populations) kept in enclosed environments (e.g., chemostats) for several generations (Fussmann et al., 2007; Maharjan et al., 2006). Modeling approaches have been limited by the tendency of the trait variance to unrealistically decline to zero over time (Merico et al., 2014; Romero‐Mujalli et al., 2019b). Our study shows that a simulation of trait dynamics more in line with developmental systems responding flexibly to internal (genotype) and external inputs (environment) enabling highly diverse functional responses, as done for saturating and sinusoidal plasticity, could help to overcome this limitation. So far, our implementation offers a phenomenological alternative only. Future theoretical work should focus on unraveling the mechanisms that make the above‐discussed phenotypic response (trait dynamics) possible. Furthermore, and in contrast to other forms of plasticity, flexible development of the phenotype can provide an adaptive response in a novel environment that need not to have been prescreened by earlier selection, which is particularly true for learning (Laland et al., 2015; Romero‐Mujalli et al., 2017; Watson & Szathmáry, 2016).

### 4.2 The relative importance of adaptive and nonadaptive phenotypic plasticity differs between life history strategies

According to our model, any form of adaptive phenotypic plasticity is of advantage over nonadaptive plasticity under controlled systematic changes of environmental conditions, as it is the case of laboratory experiments (Figure 5a). However, under stochastic environmental fluctuations typical of natural habitats, adaptive phenotypic plasticity is particularly of advantage when facing positively autocorrelated environmental fluctuations (i.e., red noise) and slow directional environmental change, and for a broader spectrum of climatic fluctuations when there is a faster trend in the mean environmental optimum (Figures 7, 8, 9). Particularly, all forms of adaptive phenotypic plasticity were superior to lack of plasticity for life history strategies with relatively slow growth rate (weak density compensation) (Figure 7). This life history strategy resembles characteristics of many vertebrate species (e.g., birds and mammals). In contrast, for life history strategies with strong density dependence effects, and therefore, fast growth rate, genetic determinism, and all forms of plasticity performed equally well (Figure 8). For these life history strategies, only under relatively high rates of directional climate change, some forms of adaptive phenotypic plasticity (i.e., linear and saturating phenotypic plasticity) and, interestingly, random nonadaptive plasticity were of advantage (Figures 8 and 9). The difference in the relative importance of adaptive and nonadaptive plasticity among life history strategies was even more evident under scenarios of genetic constraints (in our model, scenarios of relatively low mutation rate, and of slightly deleterious mutations fitness effects). Under such scenarios, life history strategies with strong density compensation could cope with the changing environmental conditions relying on random nonadaptive plasticity only (Figure 9 and Figure S2). This was not the case for life history strategies with weak density compensation, which strongly depended on adaptive plasticity for their adaptation to the local environment. Therefore, if organisms with different life history strategies, as simulated in this model, would experience equivalent environmental fluctuations and rates of directional climate change, those where breeding females are limited to few offspring are expected to experience stronger selection for the development of mechanisms of adaptive phenotypic plasticity and stronger selection to expand the limits of their plasticity mechanisms. Populations of organisms with faster growth rate can rely on nonadaptive plasticity (translating into bet‐hedging, Donaldson‐Matasci et al., 2013) for a broader range of environmental fluctuations. As linear and saturating phenotypic plasticity does not generally outperform nonadaptive plasticity, these adaptive plastic responses are not expected to evolve for life history strategies producing relatively large numbers of offspring, unless they experience relatively rapid rates of directional environmental change. A further life history parameter promoting the evolution of adaptive phenotypic plasticity is longevity/generation time, leading to a limited genetic response (Forsman, 2014, not tested in our study).

The importance of adaptive phenotypic plasticity for organisms experiencing directional change of the mean environmental optimum, as inferred in our simulations, may equally apply to dispersing and sessile organisms. Dispersing organisms may experience gradual changes in the mean environmental optimum and will benefit from developing adaptive forms of phenotypic plasticity as they expand their range, especially if density dependence is weak, as it occurs in mammal and bird species. Similarly, sessile organisms exposed to seasonal changes in the mean environmental optimum will also benefit from adaptive forms of phenotypic plasticity, particularly if their longevity is high (Borges, 2008). Examples are plant species inhabiting temperate regions (Chmielewski & Rötzer, 2001), as well as plants experiencing regular yearly cycles of rain, drought, and fire at the equator (Fajardo et al., 2005).

Despite of their good performance across scenarios of directional stochastic environmental change (as tested in this study), populations with strong density dependence are susceptible to extinction under stochastic fluctuations in environmental quality (i.e., stochastic carrying capacity) (Figure 10). Fluctuations in the carrying capacity can result from fire, resource contamination, or human land use, among other factors (Anderson et al., 2015). In contrast, populations with weak density compensation are less impacted by fluctuations in the carrying capacity of their environment. This would, for example, suggest that insect populations are more impacted by fluctuations in habitat quality (e.g., because of land use) than mammals. It remains however to be investigated, in how far this observation depends on the specific model implementation.

### 4.3 Phenotypic plasticity and environmental noise: When adaptive plasticity hinders evolution

To date, most studies of phenotypic plasticity (and its evolution) under stochastic environmental fluctuations have been focused on the level of correlation among the environmental optimum and the environmental cues sensed by organisms (Ashander et al., 2016; Ergon & Ergon, 2016; Reed et al., 2010). They show that adaptive phenotypic plasticity can only evolve under positive correlation of cues with the environmental optimum (environmental predictability). It is important to note that this type of predictability is not the same as the predictable (red noise) year‐to‐year pattern of our simulations. Specifically, this study adds that adaptive forms of phenotypic plasticity can decrease extinction risk under positively autocorrelated environmental stochasticity (red noise) (Figures 7, 8; Figure S3), while extinction risk is expected to be high in the absence of plasticity (Mustin et al., 2013). However, if the environment changes after the sensitive period when the trait is susceptible to be modified by plasticity, those traits initially developed by adaptive plasticity can lead to a disadvantage under negatively autocorrelated environmental stochasticity (blue noise) (Fig. S3), which agrees with results from evolution experiments (Hallsson & Björklund, 2012). This applies for constant characters, that is, phenotypic traits plastic early in ontogeny, resulting in mature phenotypes which cannot be further modified by the environment (Lande, 2014; Romero‐Mujalli et al., 2019b). In our model, such disadvantage of adaptive plasticity under blue noise occurred only for life history strategies with weak density compensation (Figure S3). Whether the plastic response precedes or follows the environmental change seems to have little impact under life history strategies with stronger density compensation (Figure S3). Thus, for life history strategies with weak density compensation under blue noise, adaptive phenotypic plasticity amplifies phenotype–environment mismatches due to the negative autocorrelation in the environmental stochasticity and, therefore, hinders evolution in the long run, while nonadaptive phenotypic plasticity and genetic determinism increase population persistence under such scenarios. Therefore, life history strategies with weak density compensation relying on any form of adaptive phenotypic plasticity under blue stochastic environmental conditions are expected to be more vulnerable—than those with stronger density compensation—unless they are able to adjust their plasticity strategy. However, adaptive plasticity (particularly, linear, and saturating phenotypic plasticity) becomes of advantage for all conditions of environmental stochasticity—even when the environmental change occurs after the sensitive period—when the rate of environmental change is relatively rapid (Figures 7, 8; Figure S3). In our model, rapid rates of directional change of 0.03 and 0.04 change the environment beyond the range captured by the stochasticity (considering a two‐sigma effect, 95%) of the original reference environment after 100 and 75 generations (or years), respectively. A “fast” rate of environmental change is not necessarily an absolute measurement. It should be related to characteristics—life history strategy—of the population, as shown in this study, and to the strength of selection (Burger & Lynch, 1995).

## 5 CONCLUSIONS

Adaptive phenotypic plasticity promotes population persistence under positively autocorrelated (red noise) environmental stochasticity and slow climate change, and for a broader range of fluctuations when the rate of directional change is faster. This form of plasticity is particularly important for life history strategies in which breeding females have a limited number of offspring (low fecundity, and hence slow population growth rate, typical of many vertebrate species: birds and mammals). Organisms producing more offspring per female may cope with environmental fluctuations relying only on genetic changes or random plasticity, unless the rate of environmental change is relatively high. Whenever plasticity precedes the environmental change, typical of constant characters, those life history strategies with weak density compensation will experience high risk of extinction in bluish habitats, unless they can adjust their plasticity into a bet‐hedging strategy.

Models employing linear reaction norms may overestimate persistence, if they do not consider limits of plasticity. Furthermore, limits to plasticity lead to genetic accommodation when costs are negligible and to the exposure of cryptic genetic variation when the plastic response is pushed toward the limits of plasticity. In addition, the modeling of plasticity as developmental systems relying on genotypes interacting flexibly with their environment enables coexistence of polymorphisms and highly diverse functional responses, leading to highly maintained genetic variation, many‐to‐one genotype–phenotype map, and to developmental canalization at the population level.

In this work, the mechanisms that shape the limits of adaptive plasticity were not explicitly modeled. Empirical work is needed to unravel molecular mechanisms that may dictate the limits of plastic responses.

## CONFLICT OF INTEREST

None.

## AUTHOR CONTRIBUTION

**Daniel Romero‐Mujalli:** Conceptualization (equal); Data curation (equal); Formal analysis (equal); Investigation (equal); Methodology (equal); Project administration (equal); Resources (equal); Software (equal); Validation (equal); Visualization (equal); Writing‐original draft (equal); Writing‐review & editing (equal). **Markus Rochow:** Data curation (equal); Investigation (equal); Validation (equal). **Sandra Kahl:** Conceptualization (equal); Visualization (equal). **Sofia Paraskevopoulou:** Conceptualization (equal); Visualization (equal). **Remco Folkertsma:** Conceptualization (equal); Visualization (equal). **Florian Jeltsch:** Conceptualization (equal); Formal analysis (equal); Funding acquisition (equal); Investigation (equal); Methodology (equal); Project administration (equal); Resources (equal); Supervision (equal); Validation (equal); Visualization (equal); Writing‐original draft (equal); Writing‐review & editing (equal). **Ralph Tiedemann:** Conceptualization (equal); Formal analysis (equal); Funding acquisition (equal); Investigation (equal); Methodology (equal); Project administration (equal); Resources (equal); Supervision (equal); Validation (equal); Visualization (equal); Writing‐original draft (equal); Writing‐review & editing (equal).

## Supporting information

Appendix S1

Appendix S2

## Data Availability

Model simulation data: Dryad https://doi.org/10.5061/dryad.6djh9w113. Model: GitHub repository: https://github.com/danielrm84/PanModel33.

## References

[ece37485-bib-0001] Aguilar‐Rodríguez, J. , Peel, L. , Stella, M. , Wagner, A. , & Payne, J. L. (2018). The architecture of an empirical genotype‐phenotype map. Evolution, 72, 1242–1260. 29676774 10.1111/evo.13487 PMC6055911

[ece37485-bib-0002] Ahnert, S. E. (2017 ). Structural properties of genotype‐phenotype maps. Journal of the Royal Society Interface, 14, 20170275. 10.1098/rsif.2017.0275 PMC5550974 28679667

[ece37485-bib-0003] Anderson, C. , Jovanoski, Z. , Towers, I. , & Sidhu, H. (2015). A simple population model with a stochastic carrying capacity. In T. Weber , M. J. McPhee , & R. S. Anderssen (Eds.), MODSIM2015, 21st International Congress on Modelling and Simulation (pp. 490–496). Modelling and Simulation Society of Australia and New Zealand. 10.36334/MODSIM.2015.A1.Anderson

[ece37485-bib-0004] Araújo, C. V. M. , Rodríguez, E. N. V. , Salvatierra, D. , Cedeño‐Macias, L. A. , Vera‐Vera, V. C. , Moreira‐Santos, M. , & Ribeiro, R. (2016). Attractiveness of food and avoidance from contamination as conflicting stimuli to habitat selection by fish. Chemosphere, 163, 177–183. 27526061 10.1016/j.chemosphere.2016.08.029

[ece37485-bib-0005] Araújo, C. V. M. , Shinn, C. , Moreira‐Santos, M. , Lopes, I. , Espíndola, E. L. G. , & Ribeiro, R. (2014). Copper‐driven avoidance and mortality in temperate and tropical tadpoles. Aquatic Toxicology, 146, 70–75. 24291082 10.1016/j.aquatox.2013.10.030

[ece37485-bib-0006] Ashander, J. , Chevin, L.‐M. , & Baskett, M. L. (2016). Predicting evolutionary rescue via evolving plasticity in stochastic environments. Proceedings of the Royal Society B‐Biological Sciences, 283, 20161690. 10.1098/rspb.2016.1690 PMC5046909 27655762

[ece37485-bib-0007] Bell, G. (2013). Evolutionary rescue and the limits of adaptation. Philosophical Transactions of the Royal Society B: Biological Sciences, 368, 20120080. 10.1098/rstb.2012.0080 PMC3538447 23209162

[ece37485-bib-0008] Björklund, M. , Ranta, E. , Kaitala, V. , Bach, L. A. , Lundberg, P. , & Stenseth, N. (2009). Quantitative trait evolution and environmental change. PLoS One, 4, e4521. 19229330 10.1371/journal.pone.0004521 PMC2639695

[ece37485-bib-0009] Boettiger, C. , Ross, N. , & Hastings, A. (2013). Early warning signals: The charted and uncharted territories. Theoretical Ecology, 6, 255–264.

[ece37485-bib-0010] Borges, R. M. (2008). Plasticity comparisons between plants and animals: Concepts and mechanisms. Plant Signaling & Behavior, 3, 367–375. 19513224 10.4161/psb.3.6.5823 PMC2634305

[ece37485-bib-0011] Botero, C. A. , Weissing, F. J. , Wright, J. , & Rubenstein, D. R. (2015). Evolutionary tipping points in the capacity to adapt to environmental change. Proceedings of the National Academy of Sciences of the United States of America, 112, 184. 25422451 10.1073/pnas.1408589111 PMC4291647

[ece37485-bib-0012] Burger, R. , & Lynch, M. (1995). Evolution and extinction in a changing environment: A quantitative‐genetic analysis. Evolution, 49, 151–163. 28593664 10.1111/j.1558-5646.1995.tb05967.x

[ece37485-bib-0013] Chevin, L.‐M. , Lande, R. , & Mace, G. M. (2010). Adaptation, plasticity, and extinction in a changing environment: Towards a predictive theory. PLoS Biology, 8, e1000357. 20463950 10.1371/journal.pbio.1000357 PMC2864732

[ece37485-bib-0014] Chmielewski, F.‐M. , & Rötzer, T. (2001). Response of tree phenology to climate change across Europe. Agricultural & Forest Meteorology, 108, 101–112.

[ece37485-bib-0073] DeWitt, T. J. , Sih, A. , & Wilson, D. S. (1998). Costs and limits of phenotypic plasticity. Trends Ecology & Evolution, 13(2), 77–81. 10.1016/s0169-5347(97)01274-3 21238209

[ece37485-bib-0015] Donaldson‐Matasci, M. C. , Bergstrom, C. T. , & Lachmann, M. (2013). When unreliable cues are good enough. American Naturalist, 182, 313–327. 10.1086/671161 23933723

[ece37485-bib-0016] Ergon, T. , & Ergon, R. (2016). When three traits make a line: Evolution of phenotypic plasticity and genetic assimilation through linear reaction norms in stochastic environments. Journal of Evolutionary Biology, 30, 486–500. 27862551 10.1111/jeb.13003

[ece37485-bib-0017] Eyre‐Walker, A. , Keightley, P. D. , Smith, N. G. C. , & Gaffney, D. (2002). Quantifying the slightly deleterious mutation model of molecular evolution. Molecular Biology and Evolution, 19, 2142–2149. 12446806 10.1093/oxfordjournals.molbev.a004039

[ece37485-bib-0018] Fajardo, L. , González, V. , Nassar, J. M. , Lacabana, P. , Portillo Q., C. A. , Carrasquel, F. , & Rodríguez, J. P. (2005). Tropical dry forests of Venezuela: Characterization and current conservation status. Biotropica, 37, 531–546.

[ece37485-bib-0019] Forsman, A. (2014). Rethinking phenotypic plasticity and its consequences for individuals, populations and species. Heredity, 115, 276. 25293873 10.1038/hdy.2014.92 PMC4815454

[ece37485-bib-0020] Franks, S. J. , Weber, J. J. , & Aitken, S. N. (2014). Evolutionary and plastic responses to climate change in terrestrial plant populations. Evolutionary Applications, 7, 123–139. 24454552 10.1111/eva.12112 PMC3894902

[ece37485-bib-0021] Fussmann, G. F. , Loreau, M. , & Abrams, P. A. (2007). Eco‐evolutionary dynamics of communities and ecosystems. Functional Ecology, 21, 465–477.

[ece37485-bib-0022] García‐Carreras, B. , & Reuman, D. C. (2011). An empirical link between the spectral colour of climate and the spectral colour of field populations in the context of climate change. Journal of Animal Ecology, 80, 1042–1048. 10.1111/j.1365-2656.2011.01833.x 21466552

[ece37485-bib-0023] Gonzalez, A. , Ronce, O. , Ferriere, R. , & Hochberg, M. E. (2013). Evolutionary rescue: An emerging focus at the intersection between ecology and evolution. Philosophical Transactions of the Royal Society B: Biological Sciences, 368, 20120404. 10.1098/rstb.2012.0404 PMC3538460 23209175

[ece37485-bib-0024] Grimm, V. , Berger, U. , Bastiansen, F. , Eliassen, S. , Ginot, V. , Giske, J. , Goss‐Custard, J. , Grand, T. , Heinz, S. K. , Huse, G. , Huth, A. , Jepsen, J. U. , Jørgensen, C. , Mooij, W. M. , Müller, B. , Pe'er, G. , Piou, C. , Railsback, S. F. , & Robbins, A. M. , … DeAngelis, D. L. (2006). A standard protocol for describing individual‐based and agent‐based models. Ecological Modelling, 198, 115–126.

[ece37485-bib-0025] Grimm, V. , Berger, U. , DeAngelis, D. L. , Polhill, J. G. , Giske, J. , & Railsback, S. F. (2010). The ODD protocol: A review and first update. Ecological Modelling, 221, 2760–2768.

[ece37485-bib-0026] Hallsson, L. R. , & Björklund, M. (2012). Selection in a fluctuating environment leads to decreased genetic variation and facilitates the evolution of phenotypic plasticity. Journal of Evolutionary Biology, 25, 1275–1290. 22519748 10.1111/j.1420-9101.2012.02512.x

[ece37485-bib-0027] Jordan, P. J. , & Deaton, L. E. (1999). Osmotic regulation and salinity tolerance in the freshwater snail *Pomacea bridgesi* and the freshwater clam Lampsilis teres. Comparative Biochemistry and Physiology Part A Molecular Integrative Physiology, 122, 199–205.

[ece37485-bib-0028] Klausmeier, C. A. , Osmond, M. M. , Kremer, C. T. , & Litchman, E. (2020). Ecological limits to evolutionary rescue. Philosophical Transactions of the Royal Society of London. Series B, Biological Sciences, 375, 20190453. 33131439 10.1098/rstb.2019.0453 PMC7662203

[ece37485-bib-0029] Kopp, M. , & Matuszewski, S. (2014). Rapid evolution of quantitative traits: Theoretical perspectives. Evolutionary Applications, 7, 169–191. 24454555 10.1111/eva.12127 PMC3894905

[ece37485-bib-0030] Kulkarni, S. S. , Denver, R. J. , Gomez‐Mestre, I. , & Buchholz, D. R. (2017). Genetic accommodation via modified endocrine signalling explains phenotypic divergence among spadefoot toad species. Nature Communications, 8, 993. 10.1038/s41467-017-00996-5 PMC5648835 29051478

[ece37485-bib-0031] Laakso, J. , Kaitala, V. , & Ranta, E. (2001). How does environmental variation translate into biological processes? Oikos, 92, 119–122.

[ece37485-bib-0032] Laakso, J. , Kaitala, V. , & Ranta, E. (2004). Non‐linear biological responses to environmental noise affect population extinction risk. Oikos, 104, 142–148.

[ece37485-bib-0033] Laland, K. N. , Uller, T. , Feldman, M. W. , Sterelny, K. , Müller, G. B. , Moczek, A. , Jablonka, E. , & Odling‐Smee, J. (2015). The extended evolutionary synthesis: Its structure, assumptions and predictions. Proceedings of the Royal Society B, 282, 20151019. 26246559 10.1098/rspb.2015.1019 PMC4632619

[ece37485-bib-0034] Lande, R. (2009). Adaptation to an extraordinary environment by evolution of phenotypic plasticity and genetic assimilation. Journal of Evolutionary Biology, 22, 1435–1446. 19467134 10.1111/j.1420-9101.2009.01754.x

[ece37485-bib-0035] Lande, R. (2014). Evolution of phenotypic plasticity and environmental tolerance of a labile quantitative character in a fluctuating environment. Journal of Evolutionary Biology, 27, 866–875. 24724972 10.1111/jeb.12360

[ece37485-bib-0036] Levis, N. A. , & Pfennig, D. W. (2020). Plasticity‐led evolution: A survey of developmental mechanisms and empirical tests. Evolution & Development, 22, 71–87. 31449722 10.1111/ede.12309

[ece37485-bib-0037] Lynch, M. , & Walsh, B. (1998). Genetics and analysis of quantitative traits. Sinauer Associates. https://books.google.de/books?id=UhCCQgAACAAJ

[ece37485-bib-0038] Maharjan, R. , Seeto, S. , Notley‐McRobb, L. , & Ferenci, T. (2006). Clonal adaptive radiation in a constant environment. Science, 313, 514. 16825532 10.1126/science.1129865

[ece37485-bib-0039] Martin, G. , Lenormand, T. , & Goodnight, C. (2006). The fitness effect of mutations across environments: A survey in light of fitness landscape models. Evolution, 60, 2413–2427. 17263105

[ece37485-bib-0040] Merico, A. , Brandt, G. , Smith, S. L. , & Oliver, M. (2014). Sustaining diversity in trait‐based models of phytoplankton communities. Frontiers in Ecology and Evolution, 2, 59.

[ece37485-bib-0041] Murren, C. J. , Auld, J. R. , Callahan, H. , Ghalambor, C. K. , Handelsman, C. A. , Heskel, M. A. , Kingsolver, J. G. , Maclean, H. J. , Masel, J. , Maughan, H. , Pfennig, D. W. , Relyea, R. A. , Seiter, S. , Snell‐Rood, E. , Steiner, U. K. , & Schlichting, C. D. (2015). Constraints on the evolution of phenotypic plasticity: Limits and costs of phenotype and plasticity. Heredity, 115, 293–301. 25690179 10.1038/hdy.2015.8 PMC4815460

[ece37485-bib-0042] Mustin, K. , Dytham, C. , Benton, T. G. , & Travis, J. M. J. (2013). Red noise increases extinction risk during rapid climate change. Diversity and Distributions, 19, 815–824.

[ece37485-bib-0043] Ohta, T. (1973). Slightly deleterious mutant substitutions in evolution. Nature, 246, 96. 4585855 10.1038/246096a0

[ece37485-bib-0044] Paaby, A. B. , & Rockman, M. V. (2014). Cryptic genetic variation: Evolution’s hidden substrate. Nature Reviews Genetics, 15, 247. 10.1038/nrg3688 PMC4737706 24614309

[ece37485-bib-0045] Parsons, K. J. , McWhinnie, K. , Pilakouta, N. , & Walker, L. (2020). Does phenotypic plasticity initiate developmental bias? Evolution & Development, 22, 56–70. 31348849 10.1111/ede.12304 PMC7004013

[ece37485-bib-0046] Payne, J. L. , & Wagner, A. (2019). The causes of evolvability and their evolution. Nature Reviews Genetics, 20, 24–38. 10.1038/s41576-018-0069-z 30385867

[ece37485-bib-0047] Pigliucci, M. (2005). Evolution of phenotypic plasticity: Where are we going now? Trends in Ecology & Evolution, 20, 481–486. 16701424 10.1016/j.tree.2005.06.001

[ece37485-bib-0048] Posadas, D. M. , & Carthew, R. W. (2014). MicroRNAs and their roles in developmental canalization. Current Opinion in Genetics & Development, 27, 1–6. 24791686 10.1016/j.gde.2014.03.005 PMC4125612

[ece37485-bib-0049] Reed, T. E. , Waples, R. S. , Schindler, D. E. , Hard, J. J. , & Kinnison, M. T. (2010). Phenotypic plasticity and population viability: The importance of environmental predictability. Proceedings of the Royal Society B: Biological Sciences, 277, 3391–3400. 10.1098/rspb.2010.0771 PMC2982227 20554553

[ece37485-bib-0050] Reusch, T. B. H. (2014). Climate change in the oceans: Evolutionary versus phenotypically plastic responses of marine animals and plants. Evolutionary Applications, 7, 104–122. 24454551 10.1111/eva.12109 PMC3894901

[ece37485-bib-0051] Romero‐Mujalli, D. , Cappelletto, J. , Herrera, E. A. , & Tárano, Z. (2017). The effect of social learning in a small population facing environmental change: An agent‐based simulation. Journal of Ethology, 35, 61–73.

[ece37485-bib-0052] Romero‐Mujalli, D. , Jeltsch, F. , & Tiedemann, R. (2019a). Elevated mutation rates are unlikely to evolve in sexual species, not even under rapid environmental change. BMC Evolutionary Biology, 19, 175. 31462290 10.1186/s12862-019-1494-0 PMC6714099

[ece37485-bib-0053] Romero‐Mujalli, D. , Jeltsch, F. , & Tiedemann, R. (2019b). Individual‐based modeling of eco‐evolutionary dynamics: State of the art and future directions. Regional Environmental Change, 19, 1–12.

[ece37485-bib-0054] Scheiner, S. M. (1993). Genetics and evolution of phenotypic plasticity. Annual Review of Ecology and Systematics, 24, 35–68.

[ece37485-bib-0055] Scheiner, S. M. , Barfield, M. , & Holt, R. D. (2017). The genetics of phenotypic plasticity. XV. Genetic assimilation, the Baldwin effect, and evolutionary rescue. Ecology and Evolution, 7, 8788–8803. 29152178 10.1002/ece3.3429 PMC5677470

[ece37485-bib-0056] Scheiner, S. M. , Barfield, M. , & Holt, R. D. (2020). The genetics of phenotypic plasticity. XVII. Response to climate change. Evolutionary Applications, 13, 388–399. 31993084 10.1111/eva.12876 PMC6976953

[ece37485-bib-0057] Schlichting, C. D. , & Wund, M. A. (2014). Phenotypic plasticity and epigenetic marking: An assessment of evidence for genetic accommodation. Evolution, 68, 656–672. 24410266 10.1111/evo.12348

[ece37485-bib-0058] Schwager, M. , Johst, K. , & Jeltsch, F. (2006). Does Red noise increase or decrease extinction risk? Single extreme events versus series of unfavorable conditions. American Naturalist, 167, 879–888. 10.1086/503609 16615033

[ece37485-bib-0059] Solan, M. , & Whiteley, N. (2016). Stressors in the Marine Environment: Physiological and ecological responses; societal implications. Oxford University Press. 10.1093/acprof:oso/9780198718826.001.0001

[ece37485-bib-0060] Sommer, R. J. (2020). Phenotypic plasticity: From theory and genetics to current and future challenges. Genetics, 215, 1. 32371438 10.1534/genetics.120.303163 PMC7198268

[ece37485-bib-0061] van Gestel, J. , & Weissing, F. J. (2016). Regulatory mechanisms link phenotypic plasticity to evolvability. Scientific Reports, 6, 24524. 27087393 10.1038/srep24524 PMC4834480

[ece37485-bib-0062] Vasseur, D. A. , & Yodzis, P. (2004). The color of environmental noise. Ecology, 85, 1146–1152.

[ece37485-bib-0063] Via, S. , Gomulkiewicz, R. , Jong, G. D. , Scheiner, S. M. , Schlichting, C. D. , & Tienderen, P. H. V. (1995). Adaptive phenotypic plasticity: Consensus and controversy. Trends in Ecology & Evolution, 10, 212–217. 21237012 10.1016/s0169-5347(00)89061-8

[ece37485-bib-0064] Vincenzi, S. (2014). Extinction risk and eco‐evolutionary dynamics in a variable environment with increasing frequency of extreme events. Journal of the Royal Society, Interface, 11, 20140441. 10.1098/rsif.2014.0441 PMC4208378 24920116

[ece37485-bib-0065] Wagner, A. (2008). Robustness and evolvability: A paradox resolved. Proceedings of the Royal Society B: Biological Sciences, 275, 91–100. 10.1098/rspb.2007.1137 PMC2562401 17971325

[ece37485-bib-0066] Wagner, A. (2005). Robustness and evolvability in living systems. Princeton University Press.

[ece37485-bib-0067] Watson, R. A. , & Szathmáry, E. (2016). How can evolution learn? Trends in Ecology & Evolution, 31, 147–157. 26705684 10.1016/j.tree.2015.11.009

[ece37485-bib-0068] West‐Eberhard, M. J. (2003). Developmental plasticity and evolution. Oxford University Press.

[ece37485-bib-0069] Wiens, J. J. (2016). Climate‐related local extinctions are already widespread among plant and animal species. PLoS Biology, 14, e2001104. 27930674 10.1371/journal.pbio.2001104 PMC5147797

[ece37485-bib-0070] Wiesenthal, A. A. , Müller, C. , & Hildebrandt, J.‐P. (2018). Potential modes of range shifts in euryhaline snails from the Baltic Sea and fresh water lakes in northern Germany. Hydrobiologia, 811, 339–350.

[ece37485-bib-0071] Wilensky, U. (1999). NETLOGO. Centre for Connected Learning and Computer‐Based Modelling, Northwestern University, Evanston, IL. http://ccl.northwestern.edu/netlogo. Accessed 19 March 2021.

[ece37485-bib-0072] Wilson, D. , & Cooper, J. P. (1969). Effect of light intensity during growth on leaf anatomy and subsequent light‐saturated photosynthesis among contrasting lolium genotypes. New Phytologist, 68, 1125–1135.

