# Supplementary material for: Adaptive and nonadaptive plasticity in changing environments: Implications for sexual species with different life history strategies
Source: Ecol Evol. 2021 Apr 4;11(11):6341–57. doi: 10.1002/ece3.7485 (PMC8207414; doi:10.1002/ece3.7485)
Supplement: Supplementary file 1 — Appendix S1 [file ECE3-11-6341-s001.docx]

ODD protocol

PanModel33: an eco-evolutionary model of adaptation to local environmental conditions

***i*) Purpose**

The purpose of the model is to study the ability of different kinds of populations of organisms or life strategies to adapt to different scenarios of environmental change. The model was programmed in Netlogo and designed for hypothesis testing, theory development and for communication and learning. A manual explaining how to install and use the model can be found on GitHub (<https://github.com/danielrm84/PanModel33>).

***ii*) Entities, state variables and scales**

What kinds of entities are in the model?

There are two kinds of entities: individuals or turtles (in Netlogo, individuals are called turtles), and patches describing the environment.

By what state variables, or behavioral attributes, are these entities characterized?

Turtles are characterized by sex, stage (whether adult or juvenile), fitness, fecundity, and their phenotype with its genetic and environmental components.

Patches contain two state variables: the mean environmental optimum, which is the optimum phenotype as given by the environment, and its environmental variance. Patches also contain variables recording the degree of maladaptation of the population, and whether extinction occurred, and if “true”, at what time (in generations).

The model has two modules: the Ecology and the Evolution modules. The former governs how the phenotype of organisms interacts with the environment; the latter, how the phenotype is produced. In the Ecology module, the study organism is further characterized by two global parameters: one governing whether it is a specialist, moderate or generalist organism (in our simulations we only considered moderate organisms); and the other, affecting the strength of the density dependence effect, which impacts on the resulting population dynamics (e.g., *r* or *K* strategists). In the Evolution module, global parameters define the number of loci, heritability (only works for standard model with no phenotypic plasticity), initial genetic variance, mutation probability, and mutation effect-size. The Evolution module also includes phenotypic plasticity which can be set to: no-plasticity (genetic determinism: phenotype determined by genetic component only), random, linear reaction-norm, or adaptive (with two shapes, saturating and sinusoidal).

What are the temporal and spatial resolutions and extents of the model?

The model is spatial explicit (2D), though patches are currently equivalent in terms of the environmental state. In our work we focused on local adaptation, thus, space to our model was spatially implicit. However, the model is implemented such that it can facilitate future simulations of spatial heterogeneity (e.g., patches differing in quality). The time is discrete, and each time step is a generation. By default, simulations last for 100 generations. The user can modify this parameter value in the user interface.

***iii*) Process overview and scheduling**

What entity does what and in what order?

The iterative loop each generation runs according to the following pseudo-code:

1. ask turtles [ update-phenotype ]
2. ask turtles

[

set fitness

set fecundity

]

1. ask turtles [ reproduce ]
2. Observer: < adults die >
3. Observer: < check extinction >
4. ask patches [ update-optimum ]
5. Observer: < repeat the loop >

This scheme is also shown in Fig 1 of the manuscript (flowchart).

***iv*) Design concepts**

- Emergence

What emerge from the model (rather than being imposed)?

The adaptive response of the population emerges from the model. Population-level attributes as the heritability of the evolving trait and the additive genetic variance can also emerge, rather than being imposed, depending on the chosen experimental setup. An important model output is whether the population goes extinct or can sustain the simulated scenario of environmental change. After considering several replications, this result can lead to the calculation of the probability of persistence (see the Methods section of the manuscript).

- Adaptation

How do agents adapt to improve their fitness (directly and indirectly)?

Turtles can adapt to the environment by means of genetic changes (evolution). Plasticity, conceived as a non-genetic environmentally induced phenotypic response, can also affect the ability of organisms to adapt to their local environment. Changes in parameters values governing the genetic properties and the plasticity of organisms, can affect their ability to adapt to the simulated environmental conditions.

- Fitness

What are the goals of the agent (or turtle)? What determines its survival?

The closer a turtle is to the environmental optimum, the higher its degree of adaptation (fitness proxy). Agents that perform well in their environment can contribute with more offspring for the next generation (higher fecundity). The fecundity is affected by density dependence effects. Currently only limits to phenotypic plasticity are considered. Costs of plasticity are not yet implemented. One alternative could be to implement costs to plasticity in terms of fecundity costs.

- Prediction

How do agents predict the consequences of their decisions? Use of learning, memory, environmental cues, embedded assumptions

The concept of prediction is not explicitly considered. There is no learning in the model. Though it can be implemented into the functions of phenotypic plasticity.

- Sensing

What are agents assumed to know or perceive when making decisions?

Is the sensing process itself explicitly modeled?

The sensing process itself is not explicitly modeled. When phenotypic plasticity is assumed to be linear reaction norm, or adaptive, the ability of sensing the environment is assumed to be equal and reliable for all turtles.

- Interaction

What forms of interactions among agents are there?

Turtles interact indirectly via competition for resources and directly through mating. Density dependence effects affect the fecundity of the turtles. The parameter governing the carrying capacity of the environment can be modified in the user interface to allow for populations differing in the maximum size they can reach.

Turtles mate randomly with others of opposite sex, and each couple pass on its genetic contribution to its offspring. Variations in the genetic material occur by recombination and mutations.

- Stochasticity

Justification for any stochasticity in the model.

Stochasticity plays a role in setting the environmental state, in reproduction and in the process of inheritance, and when plasticity is random. The environmental optimum changes every time step according to a given rate of change *η*. The user can select among different kinds of noise color governing the stochasticity of the environment (blue, white, and red noise). The user can also select to use a deterministic environment. During reproduction the number of offspring is randomly drawn from a Poisson distribution. The process of inheritance is stochastic through the processes of mutation and recombination.

How are stochastic processes (based on pseudorandom numbers) used in the model and why?

**Environmental stochasticity.** The environmental state *θ_t_* can be stochastic according to the chosen noise color and level of autocorrelation *α* (Schwager et al. 2006):

*θ_t_ = θ_0_ + η _t_* directional change

Where *θ_0_* is the initial environmental optimum (when *t = 0*) and *η* is the rate of environmental change. Stochasticity according to colored noise is implemented as follows:

*θ^*^_t_ = θ_t_ + ϕ_t_* directional change with noise *ϕt* and stochastic environmental optimum *θ^*^_t_*.

*ϕ_t_ = αϕ_t-1_ + βξ_t_*

The autocorrelation coefficient *α* indicates the level of environmental autocorrelation and therefore the noise color: *-1 < α < 0*, blue noise; *α = 0*, white noise, and *0 < α < 1*, red noise.

The parameter *β* determines the environmental variance,

*β = σ*$\sqrt{1-\alpha^{2}}$, as in Schwager et al. (2006), where *σ^2^* is the environmental variance (input parameter).

The parameter *ξ_t_* is a random value, normally distributed with zero mean and unity of variance.

Environmental stochasticity applies for both, directional trend of the mean environment (climate change) and cyclic environmental change.

**Mating.** Sex is randomly set according to a Bernoulli distribution with probability *p = 0.5*. Individuals mate randomly (males can participate in more than one reproductive event).

**Inheritance.** In the model, the genetics is implemented according to two common methods: implicit and explicit. In our work, we used the explicit genetics method. Stochasticity operates slightly different for these two methods:

**Implicit genetics.** The genetic component of the offspring is randomly drawn from a normal distribution centered on the mean parental value, and with variance equal to half the additive genetic variance, plus the variance potentially introduced by mutation or mutational variance (Ayllón et al. 2016; Vincenzi, De Leo, and Bellingeri 2012).

**Explicit genetics**. The genetic component results from the additive allele effects of the explicitly simulated loci. Alleles values can change due to mutations and recombination. In the model individuals are diploid, each locus receiving one allele from each parent. Parental alleles are picked randomly from each locus in the chromosome (freely recombining or unlinked bi-allelic loci) (Bridle et al. 2010; Vincenzi 2014). Mutations occur randomly according to a Bernoulli distribution with probability *μ*. In case that a mutation occurs, its effect is randomly drawn from a normal distribution with zero mean and variance equals to the effect size of mutations, which is an input parameter (Vincenzi 2014). The assumption of a Gaussian distribution is consistent with analysis of mutation effects (Lynch and Walsh 1998; Martin, Lenormand, and Goodnight 2006).

**Environmental effect on the phenotype.** For the standard model, the environmental effect in the development of the trait is assumed to be random and normally distributed with zero mean and a variance *VE* that can be either fixed (input parameter) or computed as a function of the heritability and the additive genetic variance. Given the value for the narrow-sense heritability *h^2^ =* $\frac{VA}{VP}$; where *VA* and *VP* are the additive genetic variance and the phenotypic variance respectively,

*VE =* $\frac{VA}{h2}$ *- VA*

If the user decides to account for any form of phenotypic plasticity as simulated in the model, heritability is no longer constant, but an emerging property.

Are stochastic processes used,

to initialize the model?

In the initial population, the genotype of each organism is created randomly and assumed locally adapted. Implicit genetics: for each organism, the value for its genetic component is drawn from a normal distribution with mean equal to the environmental optimum and variance equal to the initial additive genetic variance. Explicit genetics: for each individual organism, alleles coding for its phenotype were drawn from a normal distribution with mean equal to the environmental optimum *θ_0_* and variance V = VG / 2L, where VG was the initial genetic variance present in the population, and L the number of loci affecting the phenotypic trait. Then, the genetic component along with the selected method for phenotypic plasticity build up the phenotype of the organism.

- Collectives

Grouping of individuals.

Collectives are not represented in the model.

- Observation

What outputs are needed to test the model and to solve the problem the model was designed for?

The model was designed to investigate on the ability of different types of organisms for their adaptation to novel local environmental conditions. This can be assessed by monitoring trends overtime in population size, degree of local adaptation, genetic and phenotypic variances, match of the population mean phenotype with the environmental optimum, and by recording extinction events. After several replications, the probability of persistence can be computed for each kind of organism and each scenario of environmental change.

What outputs from the model are needed to observe its internal dynamics as well as its system-level behavior?

The user interface already includes plots showing the internal real time dynamics of the simulation. This includes the genotypic and phenotypic distributions in the population in relation to the environmental optimum. The system-level behavior can be monitored through time series plots describing the population abundance, genetic and phenotypic variances; and by a panel monitoring the degree of adaptation of the population.

What tools (graphics, file output, data on individuals, etc.) are needed to obtain these outputs?

The user interface already includes the above mentioned plots for real time monitoring of simulation runs. Netlogo software allows exporting plot data in *.csv format for further analysis. In addition, simulation experiments that involve several replicates and systematic variations of model settings can be run using the *BehaviorSpace* tool of Netlogo. The resulting output file (*.csv) can be opened with the preferred program (e.g., R) to compute, for example, the probability of persistence over the selected time span (in generations).

***v*) Initialization**

What is the initial state of the model world? How many entities? What are the exact values of their state variables (or were they set stochastically)?

The initialization of the model world or setup, includes the following:

- The initial environmental state is set to its default initial value. This means that the patch optimum is set to zero (initial environmental optimum).
- *N* agents are created (by default *N = 1000*) and the population is assumed to be locally adapted. Therefore, the resulting mean phenotype of the population is centered on the initial environmental optimum.
- The genetic component of the phenotype is set according to the selected method of genetics (explicit or implicit).
- Phenotypic plasticity is set according to the preferred method (standard model, random, linear reaction norm, adaptive saturating or adaptive sinusoidal).
- The phenotype of the turtles (individuals) is then computed as a linear additive effect of its genetic and environmental or plastic components.

Since the initialization involves stochasticity, initial conditions can vary among simulations, especially when the population size is small.

Are the initial values chosen arbitrary or based on data? (reference to those data should be provided)

The initial values can be chosen arbitrary or can be based on data. For example, the mutation probability, mutation effect-size, number of loci, and heritability of the trait are parameter values that can be set according to data.

***vi*) Input data**

Does the model use input from external sources such as data files or other models to represent processes that change over time?

The model does not use external sources

***vii*) Submodels**

What, in detail, are the submodels that represent the processes listed in “Process overview and scheduling”?

What are the model parameters, their dimensions, and reference values?

How were submodels designed or chosen, and how were they parameterized and then tested?

- Set the initial environment

The initial environmental optimum is set to zero.

- Set the initial population

In the initial population, the phenotype of each organism is created randomly and assumed locally adapted. Explicit genetics: this means that for each organism, the value for its genetic component is drawn from a normal distribution with mean equal to the environmental optimum *θ_0_* and variance equal to the initial additive genetic variance. Explicit genetics: for each individual organism, alleles coding for its phenotype were drawn from a normal distribution with mean equal to the environmental optimum *θ_0_* and variance V = VG / 2L, where VG was the initial genetic variance present in the population, and L, the number of loci affecting the phenotypic trait. The phenotype *z* is then computed as,

*z = a + e*

where *a* and *e* are the genetic component and the environmental effect or phenotypic plasticity in the development of the trait, respectively. The value of the environmental effect *e* depends on the selected method for phenotypic plasticity. The standard model and random plasticity assume *e* to be random and normally distributed with zero mean and variance *VE* that can be either fixed (input parameter in the case of random plasticity) or computed as a function of the heritability and the additive genetic variance (standard model). Given the value for the narrow-sense heritability *h^2^ =* $\frac{VA}{VP}$; where *VA* and *VP* are the additive genetic variance and the phenotypic variance respectively,

*VE =* $\frac{VA}{h2}$ *- VA*

For the method linear reaction norm, *e = bθ_t_*; where *b* is the slope or degree of plasticity and *θ_t_* the environmental optimum at time *t* (in generations).

The two methods of adaptive phenotypic plasticity differ only in the condition that determine when the function is truncated – and therefore differed in what occurs beyond the limits – and were implemented as follows:

*e = ΩΔE*; where *Ω* is always positive and shapes the plastic response, as it is given by *Ω = sin(*│*ΔE*│*)*. The term *ΔE* indicates the amount of change with respect to the reference environment *θ^R^ = a*, such that ΔE = *θ_t_* – *θ*^R^. The parameter *a* is the genetic component of the phenotype.

For sinusoidal phenotypic plasticity, if the argument of the sine function is greater than *π*, *e = 0* (the organism fails to develop a plastic response). On the other hand, for saturating phenotypic plasticity, if the argument of the sine function is greater than *π / 2*; the term *ΔE* is set to *ΔE = π / 2* such that a maximum response is reached (saturation).

These two methods were designed based on observations from stress tolerance responses for some physiological and behavioral traits (Araújo et al. 2014; Jordan and Deaton 1999; Solan and Whiteley 2016).

- Update phenotype

This function computes (or updates) the phenotype *z* of individual *i* as an additive linear combination of its genetic and environmental or phenotypic plasticity components, *a* and *e*, respectively. Thus,

$$z=a+e$$

- Set fitness

The model assumes Gaussian stabilizing selection acting on a single quantitative character *z* with the optimum phenotype *θ_t_* exhibiting temporal change as mentioned above. The model allows for selecting between two Gaussian fitness functions that give qualitatively similar results. The strength of selection *γ* affects the width of the fitness function and its value affects the type of organism that is simulated, with specialists experiencing the strongest selection and generalists the weakest. Thus, the fitness *w* of individual *i* is given by:

Method 1 as in (Björklund et al. 2009)

$$w_{i}=1-\frac{(z_{i}-\theta_{t})^{2}}{\gamma}$$

Method 2 as in (Burger and Lynch 1995)

$$w_{i}=e^{\frac{{-(z_{i}- \theta_{t})}^{2}}{2\gamma^{2}}}$$

- Set fecundity

The fecundity of the reproductive couple is the sum of the scaled fitness values (*w’_i_*) of the two parents after considering density dependence effects. The fitness was scaled according to a Ricker-type model as in Björklund et al. (2009):

$${w'}_{i}={w_{i}e}^{\psi(1-\frac{N}{K})}$$

where *N* is the population size and *K*, the carrying capacity. The parameter *ψ* describe the strength of the density dependence effect. The higher the *ψ*, the stronger is the density dependence effect. Varying the parameter *ψ* allows simulating fundamentally different population dynamics and therefore different life strategies.

- Run reproduction

Adult individuals mate randomly with others of opposite sex, with replacement only for males (i.e., males can participate in more than one reproductive event). The fecundity of the couple *λ* is equal to the sum of the scaled fitness of the partners *i*,*j*

*λ = w’_i_ + w’_j_*

Each couple produces a number of offspring randomly drawn from a Poisson distribution with expectancy *λ*

The process of inheritance of the genetic component *a* occurs according to two common methods in the literature: implicit or explicit genetics. The user can select the preferred method in the user interface.

Method 1 implicit genetics

In this method, genetics is implicit according to the infinitesimal model of quantitative genetics (Lynch and Walsh 1998), which assumes that quantitative traits are affected by a large number of loci of additive effects. Therefore trait inheritance can be approximated using a normal distribution with mean centered on the arithmetic mean of the two parental trait values, and variance equal to half the additive genetic variance for the trait. The model allows for selecting among three common methods for the modeling of the additive genetic variance: 1) as an input parameter (Reed et al. 2011); 2) population-level (Vincenzi, De Leo, and Bellingeri 2012); or 3) family-level (additive genetic variance of parents) (Björklund et al. 2009). We decided to modify the infinitesimal model as in Vincenzi et al. (2012) in order to account for the decline of additive genetic variance and the new input from mutations. Thus, the variance *σG* of the distribution is given by

$$\sigma G=\frac{1}{2}(\sigma A+ \sigma mM)$$

where *σA* is the additive genetic variance, *σm*, the mutational variance, and *M*, the amplitude or effect size of mutations.

Method 2 explicit genetics

The genetic component *a* is determined by *L* unlinked diploid loci of additive effects within and among loci, as in Vincenzi (2014). Thus,

*a =* $\sum_{l=1}^{L} n_{l}$

where *n_l_* is the sum of allelic values at locus *l*. The number of loci *L* is an input parameter. Each offspring inherit one strain copy or haplotype from each parent. Allele values for each locus of the inherited haplotype are picked randomly from the corresponding parental locus (Recombination). Then, mutations take place with probability *μ* of mutation per locus. In case that a mutation occurs, its effect is randomly drawn from a normal distribution with zero mean and variance equals to the effect size of mutations, which is an input parameter (Vincenzi 2014). The assumption of a Gaussian distribution is consistent with analysis of mutation effects (Lynch and Walsh 1998; Martin, Lenormand, and Goodnight 2006).

The environmental component *e* of the phenotype is computed as explained above for phenotypic plasticity.

- Adults die

The model considers non-overlapping generations, and therefore, all adults die after the reproduction phase of the model.

- Check extinction

The model stops if extinction occurs, *N = 0*, before the time limit is reached.

- Update environmental optimum

The model allows for simulating directional environmental change, which imposes a trend on the mean environmental variable (climate change), and cyclic or seasonal environmental change. Seasonality was not investigated in our work, and represents a topic for future research. Stochasticity applies for both scenarios of environment. For climate change, the environmental optimum *θ_t_* changes at rate *η* per iteration. In the model, one iteration is equivalent to one generation. This corresponds to what is expected for a climatic variable as temperature in the presence of climate change (Kopp and Matuszewski 2014). The model also allows for simulating the environment as mentioned above under scenarios of increasing variance at rate *ρ* per generation. This leads to the increased frequency of extreme events, as droughts, floods, and heat waves that can also be associated to climate change (Vincenzi 2014; Vincenzi, De Leo, and Bellingeri 2012). Thus, there are two main scenarios of environmental change: climate change and cyclic environment, both with option to account for extreme events. Environmental stochasticity is further simulated adding colored noise around the mean value of the environmental optimum. Thus, the implemented scenarios of environment are:

Climate change scenario:

*θ_t_ = θ_0_ + η _t_* where *θ_0_* is the initial environmental optimum (when *t = 0*) and *η* is the rate of environmental change.

Cyclic environmental scenario:

*θ_t_ = Asin(2πt / T)*; where *A* governs the amplitude and *T* the period of the wave, and *t*, the time (in generations) (as in Bürger and Krall 2004).

The stochasticity was implemented as explained above within *Environmental stochasticity*.

References

Araújo, Cristiano V.M., Cândida Shinn, Matilde Moreira-Santos, Isabel Lopes, Evaldo L.G. Espíndola, and Rui Ribeiro. 2014. “Copper-Driven Avoidance and Mortality in Temperate and Tropical Tadpoles.” *Aquatic Toxicology* 146 (January): 70–75. https://doi.org/10.1016/j.aquatox.2013.10.030.

Ayllón, Daniel, Steven F. Railsback, Simone Vincenzi, Jürgen Groeneveld, Ana Almodóvar, and Volker Grimm. 2016. “InSTREAM-Gen: Modelling Eco-Evolutionary Dynamics of Trout Populations under Anthropogenic Environmental Change.” *Next Generation Ecological Modelling, Concepts, and Theory: Structural Realism, Emergence, and Predictions* 326 (April): 36–53. https://doi.org/10.1016/j.ecolmodel.2015.07.026.

Björklund, Mats, Esa Ranta, Veijo Kaitala, Lars A. Bach, Per Lundberg, and Nils Chr. Stenseth. 2009. “Quantitative Trait Evolution and Environmental Change.” *PLoS ONE* 4 (2): e4521. https://doi.org/10.1371/journal.pone.0004521.

Bridle, Jon R., Jitka Polechová, Masakado Kawata, and Roger K. Butlin. 2010. “Why Is Adaptation Prevented at Ecological Margins? New Insights from Individual-Based Simulations.” *Ecology Letters* 13 (4): 485–94. https://doi.org/10.1111/j.1461-0248.2010.01442.x.

Bürger, R., and C. Krall. 2004. “Quantitative-Genetic Models and Changing Environments.” In *Evolutionary Conservation Biology*, edited by R Ferrière, U. Dieckmann, and D. Couvet, 171–187. Cambridge: Cambridge University Press. doi:10.1017/CBO9780511542022.

Burger, Reinhard, and Michael Lynch. 1995. “Evolution and Extinction in a Changing Environment: A Quantitative-Genetic Analysis.” *Evolution* 49 (1): 151–63. https://doi.org/10.2307/2410301.

Jordan, Percy J., and Lewis E. Deaton. 1999. “Osmotic Regulation and Salinity Tolerance in the Freshwater Snail Pomacea Bridgesi and the Freshwater Clam Lampsilis Teres.” *Comparative Biochemistry and Physiology Part A: Molecular & Integrative Physiology* 122 (2): 199–205. https://doi.org/10.1016/S1095-6433(98)10167-8.

Kopp, Michael, and Sebastian Matuszewski. 2014. “Rapid Evolution of Quantitative Traits: Theoretical Perspectives.” *Evolutionary Applications* 7 (1): 169–91. https://doi.org/10.1111/eva.12127.

Lynch, M., and B. Walsh. 1998. *Genetics and Analysis of Quantitative Traits*. Sinauer. https://books.google.de/books?id=UhCCQgAACAAJ.

Martin, Guillaume, Thomas Lenormand, and C. Goodnight. 2006. “THE FITNESS EFFECT OF MUTATIONS ACROSS ENVIRONMENTS: A SURVEY IN LIGHT OF FITNESS LANDSCAPE MODELS.” *Evolution* 60 (12): 2413–27. https://doi.org/10.1554/06-162.1.

Reed, Thomas E., Daniel E. Schindler, Merran J. Hague, David A. Patterson, Eli Meir, Robin S. Waples, and Scott G. Hinch. 2011. “Time to Evolve? Potential Evolutionary Responses of Fraser River Sockeye Salmon to Climate Change and Effects on Persistence.” *PLOS ONE* 6 (6): e20380. https://doi.org/10.1371/journal.pone.0020380.

Romero-Mujalli, Daniel, Florian Jeltsch, and Ralph Tiedemann. 2019. “Elevated Mutation Rates Are Unlikely to Evolve in Sexual Species, Not Even under Rapid Environmental Change.” *BMC Evolutionary Biology* 19 (1): 175. https://doi.org/10.1186/s12862-019-1494-0.

Schwager, Monika, Karin Johst, Florian Jeltsch, Associate Editor: Wolf M. Mooij, and Editor: Michael C. Whitlock. 2006. “Does Red Noise Increase or Decrease Extinction Risk? Single Extreme Events versus Series of Unfavorable Conditions.” *The American Naturalist* 167 (6): 879–88. https://doi.org/10.1086/503609.

Solan, Martin, and Nia Whiteley. 2016. *Stressors in the Marine Environment: Physiological and Ecological Responses; Societal Implications*. Oxford, UK: Oxford University Press.

Vincenzi, Simone. 2014. “Extinction Risk and Eco-Evolutionary Dynamics in a Variable Environment with Increasing Frequency of Extreme Events.” *Journal of The Royal Society Interface* 11 (97). https://doi.org/10.1098/rsif.2014.0441.

Vincenzi, Simone, Giulio A. De Leo, and Michele Bellingeri. 2012. “Consequences of Extreme Events on Population Persistence and Evolution of a Quantitative Trait.” *Ecological Informatics* 8 (March): 20–28. https://doi.org/10.1016/j.ecoinf.2011.12.001.
