# Supplementary material for: Adaptive and nonadaptive plasticity in changing environments: Implications for sexual species with different life history strategies
Source: Ecol Evol. 2021 Apr 4;11(11):6341–57. doi: 10.1002/ece3.7485 (PMC8207414; doi:10.1002/ece3.7485)
Supplement: Supplementary file 2 — Appendix S2 [file ECE3-11-6341-s002.docx]

**SUPPLEMENTARY MATERIAL**

**
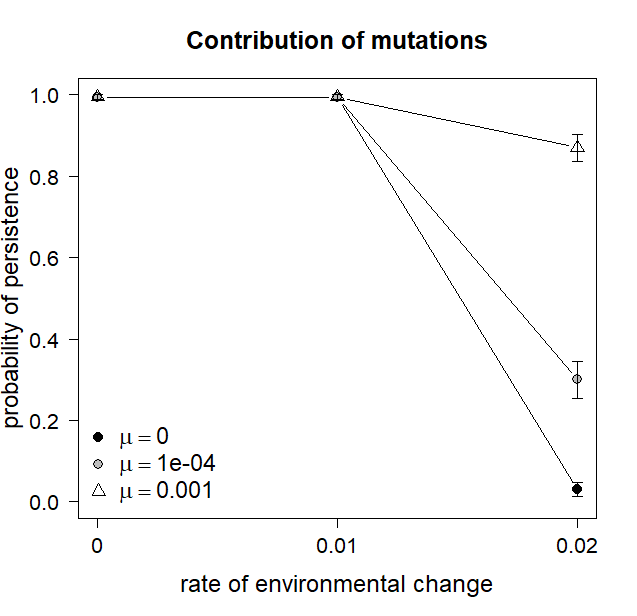
**

**Fig. S1** Contribution of mutations. Probability of persistence of a population under scenarios of no adaptation (mutation rate *μ* = 0), low (*μ* = 10^-4^) and “standard” (*μ* = 10^-3^) mutation rates experiencing stochastic (white noise) directional environmental change scenarios (rate of environmental change *η = 0*, only stochastic change, *η = 0.01* and *η = 0.02*). The standard mutation rate scenario was the most common value of mutation rate used for the results shown in the manuscript. Density compensation *ψ = 1.0*. Simulations (100 replicates) were run for 250 generations each. Error bars were estimated from the binomial distribution.

**
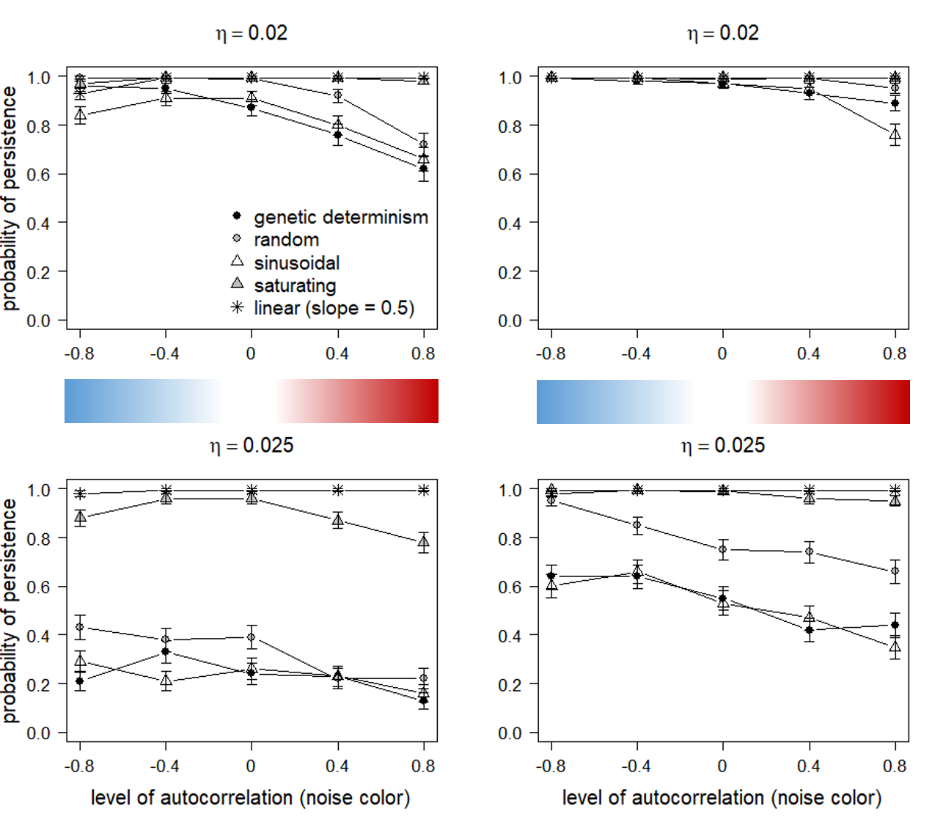
**

**Fig. S2** Relative importance of forms of non-adaptive (random) and adaptive (sinusoidal, saturating and linear) phenotypic plasticity affecting persistence of populations differing in levels of density compensation (*ψ*) and experiencing moderate rate of directional stochastic environmental change (*η = 0.02* and *0.025*, *ψ = 1.8* and *ψ = 2.5*) under scenarios of slightly deleterious mutations effects (according to Romero-Mujalli et al. 2019a). Mutation rate (*μ* = 10^-3^). A scenario of genetic determinism (narrow sense heritability *h^2^ = 1*) was also simulated. Simulations (100 replicates) were run for 250 generations each. Error bars were estimated from the binomial distribution.


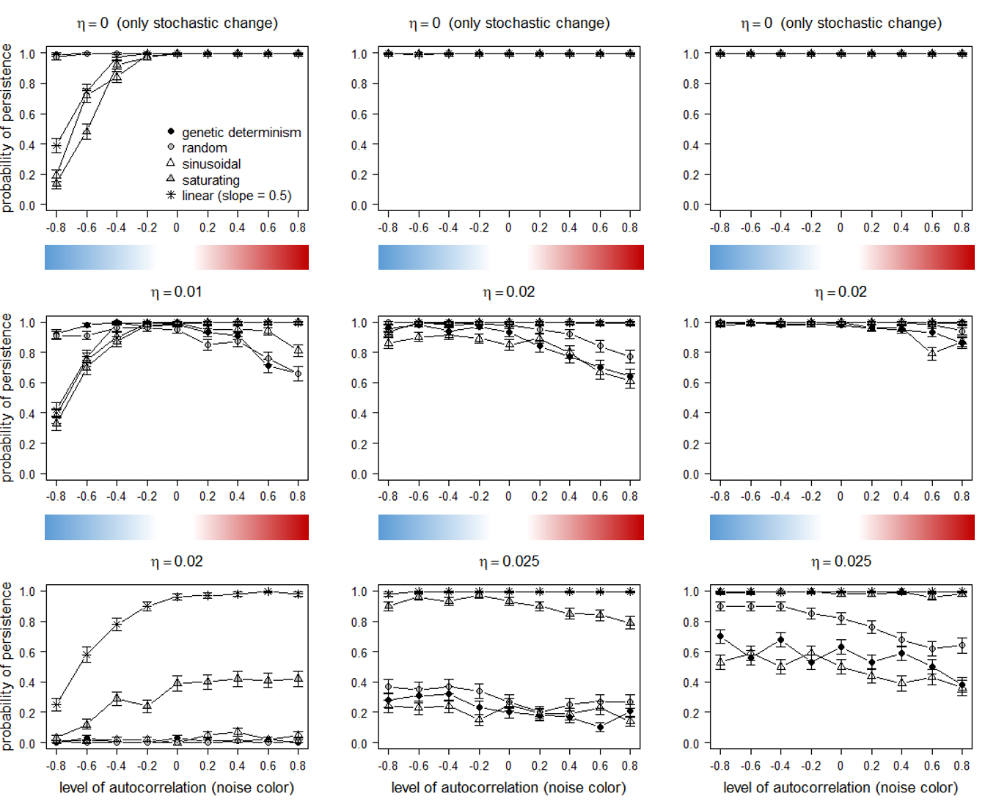


**Fig. S3** The effect of non-adaptive (random) and adaptive (linear, sinusoidal, and saturating) phenotypic plasticity on probability of persistence (100 replicates, 250 generations each) of populations with different life history strategies (*ψ = 0.5*, left column; *ψ = 1.8*, middle column; and *ψ = 2.5*, right column). Here, the environmental change occurs after the development of the matured phenotype (*i*.*e*., after a sensitive period of trait development). The linear reaction norm had a slope *b = 0.5*. A scenario of genetic determinism (narrow sense heritability *h^2^ = 1*) was also simulated. The color bar illustrates the color of the stochastic noise. For *ψ = 0.5*, *η = 0.025* was not included because most runs ended in extinction. The scenario *η = 0.01* for stronger levels of density compensation was not shown, because it did not differ from *η = 0*.
